# Intermediate filaments and their associated molecules

**DOI:** 10.7555/JBR.38.20240193

**Published:** 2025-02-08

**Authors:** Jing Gao, Fumihiko Nakamura

**Affiliations:** School of Pharmaceutical Science and Technology, Tianjin University, Tianjin 300072, China

**Keywords:** intermediate filament, intermediate filament-associated proteins, posttranslational modification, mechanotransduction, cell mechanics

## Abstract

Intermediate filaments (IFs) in human cells are the products of six distinct gene families, all sharing homology in a core rod domain. These IFs assemble into non-polar polymers, providing cytoplasmic and nuclear mechanical support. Recent research has revealed the active and dynamic properties of IFs and their binding partners. This regulation extends beyond cell mechanics to include migration, mechanotransduction, and tumor growth. Therefore, this comprehensive review aims to catalog all human IF genes and IF-associated proteins (IFAPs), detailing their names, sizes, functions, associated human diseases, relevant literature, and links to resources like UniProt and the Protein Atlas database. These links provide access to additional information such as protein structure, subcellular localization, disease-causing mutations, and pathology. Using this catalog, we will provide an overview of the current understanding of the biological functions of IFs and IFAPs. This overview is crucial for identifying gaps in their characterization and understanding IF-mediated mechanotransduction. Additionally, we will consider potential future research directions.

## Introduction

Intermediate filaments (IFs) in animal cells are one of the three major cytoskeletal filaments: microtubules (MTs, approximately 25 nm in diameter), IFs (10–12 nm), and actin filaments (approximately 6 nm). Unlike MTs and actin filaments, which are assembled from globular subunits, IFs are composed of rod-like dimers formed by a coiled-coil structure^[1–3]^. Although keratin (KRT) and neurofilaments were discovered before the term "IFs" was coined^[4–5]^, these filaments were not recognized as IFs at that time. The advent of a technique that enabled the visualization of actin filaments in cells using heavy meromyosin led to the discovery of filaments with a diameter of approximately 10 nm, which did not bind heavy meromyosin. These were termed "IFs" because their dimensions fell between those of actin filaments and the thick filaments found in striated muscle^[6]^. The proteins of intermediate-sized filaments in tissue culture cells and animal tissues were identified by immunofluorescence microscopy using specific antibodies against vimentin, keratin, desmin, and brain filament proteins^[7–8]^. This method allowed for a conclusive differentiation of IFs from MTs and actin filaments.

The main components of very stable biological structures, such as fingernails, hair, wool, horn, and the skin stratum corneum, are keratin IFs from dead epithelial cells, which are crosslinked by disulfide bonds. In contrast to these oxidized IFs, cytoplasmic IFs are dynamic and interact with other components of the cytoskeleton through IF-associated proteins (IFAPs). In epithelial cells, cytoplasmic IFs form transcellular networks through a highly ordered membrane domain called the desmosome, and they also establish connections with the extracellular matrix through hemidesmosomes. These networks are crucial for determining tissue architecture and providing mechanical resilience to cells^[9]^. In cells undergoing active proliferation and differentiation, IFs are subject to dynamic regulation, involving post-translational modifications (PTMs) and interactions with IF-binding partners.

We have recently reviewed the actin and MT cytoskeletons and cataloged their genes and associated proteins, detailing their names, sizes, functions, associated human diseases, relevant literature, and links to resources like UniProt and the Protein Atlas database^[10–11]^. However, such a catalog for IFs is unavailable, despite the existence of a number of recent reviews on IFs^[12–20]^. Using this catalog, we will provide an overview of the current understanding of the biological functions of IFs and IFAPs. This overview is essential for identifying gaps in their characterization and understanding IF-mediated mechanotransduction. Additionally, we will consider potential directions for future research. Although plant cells also express IF-like proteins^[21–22]^, they are less well-characterized and will not be described in this review article.

Given the space limitations and the relatively well-reviewed nature of the topic, an overview of the structure and functions of IFs and IFAPs is included in ***Supplementary Data*** (available online). Here, we focus on the IF-related diseases, drugs targeting IFs, the mechanics and mechanotransduction of IFs and the nucleus as well as potential future research directions.

## Diseases

Keratinopathy is associated with mutations in the keratin (types Ⅰ and Ⅱ IFs) genes^[23–25]^. To date, 16 pathogenic gene variants associated with epidermolysis bullosa (EB), a blistering skin disease, have been clearly and comprehensively reviewed^[26]^. However, the exact molecular mechanisms by which specific keratin mutations cause diseases remain unclear. Nevertheless, these mutations may compromise both structural integrity and mechanical links to hemidesmosomes/desmosomes, and affect cell signaling pathways, including those involved in inflammation and apoptosis^[27–28]^. For example, the homozygous c.1474T>C (p.S492P) mutation in the region encoding the tail domain of KRT5 is associated with epidermolysis bullosa simplex (EBS), a genetic skin disorder characterized by fragile skin that easily blisters in response to minor friction or trauma. This mutation hampers the proper assembly of keratin IFs, presumably because of a change in the peptide bond angle at the 492nd residue caused by the substitution of serine with proline, and inhibits the MAPK signaling pathways through an unknown mechanism^[29]^. Although it is not confirmed, mutations in keratin-associated proteins (KAPs) likely also cause keratinopathy, because mutations in other *KAP* genes, such as plectin, cause a form of EB, the best-studied keratinopathy^[30]^. Disruption of the IF network leads to acantholysis within the basal and suprabasal layers of the epidermis, ultimately resulting in blistering and/or hyperkeratinization of the skin^[31]^.

Interestingly, keratinopathies are not merely diseases caused by abnormalities in mechanical strength because of mutations in keratin genes; they are also heterogeneous diseases presenting multiple manifestations, ranging from epidermal fragility to epidermal hyperproliferation^[32]^.

Desminopathy is a subtype of myofibrillar myopathy caused by the loss or mutation of the desmin (type Ⅲ IF) gene and is characterized by protein aggregates accumulating in muscle fibers^[33–35]^. Heat shock proteins (HSPs), such as HSP22, HSP27, and α-crystallins, prevent protein accumulation and aggregation, and the HSP inducer, geranylgeranylacetone, may inhibit the progression of desmin-related cardiomyopathy^[36–37]^. Nevertheless, there are currently no clear and effective treatments for desminopathy; however, some complications may be prevented with early diagnosis and the use of pacemakers^[35]^.

Diseases associated with type Ⅲ IFs, including desmin, glial fibrillary acidic protein (GFAP), vimentin, peripherin, and syncoilin, may be caused by modifications by oxidants and electrophiles, because the oxidation of their conserved cysteine residues in the coil 2B domain induces structural rearrangements^[38–39]^. Therefore, type Ⅲ IFs act as sensors for oxidative and electrophilic stress. The plant poisoning vermeersiekte, which mainly occurs in sheep, is caused by several *Geigeria* species that produce sesquiterpene lactones. Sesquiterpene lactones such as ivalin and parthenolide induce aggregation of desmin^[40]^.

Loss, upregulation, or mutations of type Ⅳ IFs, such as alpha-internexin, neurofilaments, nestin, and synemin, cause various diseases^[41–43]^. Moreover, an increase in α-internexin levels is noted in certain gliomas, especially in oligodendrogliomas. Therefore, α-internexin expression appears to serve as a reliable prognostic marker^[44]^. Mutations in neurofilament genes cause several neuroaxonal and neuropsychiatric disorders characterized by disrupted subunit assembly and neurofilament aggregation^[45]^. Typically, mutations in the neurofilament light chain (*NEFL*) gene cause Charcot-Marie-Tooth disease (CMT) type 2E (CMT2E; characterized by axonal damage in the peripheral nerves, leading to muscle weakness and sensory loss), CMT1F (which involves demyelination and slowed nerve conduction), and dominant-intermediate CMT (DI-CMTG; a form with both axonal and demyelinating features). Mutations in the neurofilament heavy chain (*NEFH*) gene are associated with CMT2CC, an axonal variant of CMT that also results in progressive muscle weakness and sensory impairment in the limbs^[46–48]^. Although mutations in over 100 genes may lead to CMT, the cases related to mutations in neurofilament genes account for only a small portion of the diagnosed CMT cases^[46–49]^. CMT primarily affects the motor and sensory nerves, leading to muscle weakness and atrophy. The reported mutations in *NEFL* related to CMT are mostly missense mutations, as well as nonsense, frameshift, and deletion mutations^[50]^. However, these mutations span the entire length of *NEFL*, and their locations are independent of CMT types^[46–47]^. In contrast, mutations in *NEFH* frequently occur in the C-terminal portion of the tail domain^[46]^. Increased expression levels of nestin in melanoma are associated with an aggressive course of the disease and poor prognosis^[51]^. A mutation in the synemin gene was identified in the ulnar-mammary-like syndrome, which is associated with left ventricular tachycardia and other cardiac and skeletal myopathies^[52–53]^.

Mutations in the lens-specific type Ⅵ IF genes, beaded filament structural protein 1 and 2 (*BFSP1* and *BFSP2*), cause cataracts^[54–57]^. To better understand disease mechanisms and treatments, eye organoids have been generated; however, their application in vision research remains in its infancy^[58]^.

Laminopathies are associated with mutations in genes coding for nuclear lamins (type Ⅴ IF) and lamin-binding proteins^[59–60]^. *LMNA* is among the most mutated human genes, and its mutations lead to numerous heritable diseases^[61–62]^. For example, studies have found that the transcription factor TEA domain transcription factor 1 (TEAD1) is trapped at the nuclear membrane by mutant lamin A/C (Q353R), leading to dilated cardiomyopathy^[63–64]^. Because B-type lamins are involved in a wide range of nuclear functions, such as DNA replication and repair, as well as chromatin regulation, mutations in their genes or the dysregulation of their expression levels are critical for the onset of several diseases. For example, the duplication of *LMNB1* is associated with adult-onset leukodystrophy, and the altered LMNB1 expression leads to senescence. Mutations in *LMNB1* and *LMNB2* also cause various diseases, including neurodegenerative diseases and lipodystrophy. Moreover, mutations in the lamin-associated genes, such as lamin B receptor (*LBR*), may also cause laminopathies, including Greenberg dysplasia, which is characterized by abnormal bone development and fluid accumulation, and Pelger-Huët anomaly, a benign genetic condition marked by abnormal nuclear shapes in white blood cells^[65]^.

Importantly, IFs also interact with proteins and nucleotides from pathogens to facilitate their entry into host cells and their replication, providing a potential drug target^[66–72]^.

## Drugs acting on IFs

More than 80 human diseases are linked to mutations in genes coding for IF proteins, referred to as IF-pathies, and there are currently no available treatments that directly target IFs to address these disorders^[73]^. There are few available compounds that act on IFs, even for research purposes, compared with those available for other cytoskeletal polymers^[10–11]^. Nevertheless, owing to their diverse biological functions, some small molecules that specifically affect IFs have been discovered from natural products and synthesized. For example, retinoids and sulforaphane derived from plants have the potential to restore skin integrity by selectively increasing the expression of normal keratins (*e.g.*, KRT6 [type Ⅱ keratin], KRT16 and KRT17 [type Ⅰ keratins]). These keratins are typically expressed during wound healing or in response to skin stress. This upregulation may help compensate for the mutants associated with EBS (commonly involving mutations in *Krt5* [type Ⅱ keratin] and *Krt14* [type Ⅰ keratin]) in mouse models, highlighting the functional redundancy within the keratin family as a key factor in modulating the severity of phenotypes^[74–76]^. Studies have found that ferulic acid promotes wound healing by inducing KRT6A (type Ⅱ keratin), inhibiting beta-catenin in keratinocytes, and activating nuclear factor erythroid-2-related factor 2 at the wound edge^[77]^. Therefore, sulforaphane may be effective in addressing EBS caused by type Ⅰ keratin mutations, while ferulic acid may potentially treat EBS resulting from type Ⅱ keratin mutations. Midostaurin (https://pubchem.ncbi.nlm.nih.gov/compound/9829523) has been shown to facilitate the restoration of proper structure in IFs that incorporate mutated keratins within hepatocytes. This is achieved by enhancing their binding to a non-muscle myosin heavy chain and modifying the phosphorylation sites on keratins and desmoplakin, with the aim of treating patients affected by EBS^[78–79]^. Peptide-drug conjugates targeting KRT1 may inhibit triple-negative breast cancer in mice^[80]^. Mutations in the keratin genes associated with diseases induce PTMs in both keratins and their associated proteins, contributing to the progression of the disease. Therefore, there is a therapeutic opportunity in targeting specific PTMs and their pathways^[25]^. For instance, the expression profile signature of EBS was countered by AKT/mTOR and PI3K inhibitors. Furthermore, EBS patients undergoing topical treatment with sirolimus, an mTOR inhibitor, exhibited significant clinical improvement and a notable reduction in keratoderma^[81]^.

Aggregation of desmin (type Ⅲ) may be reduced by antioxidants, such as alpha-lipoic acid, α-tocopherol, acetyl-α-tocopherol, curcumin, and colchicine, as well as through inhibition of the Rac1 pathway (*e.g.*, NSC23766), stimulation of macroautophagy (*e.g.*, mTOR inhibitor PP242), and induction of heat shock proteins (*e.g.*, geldanamycin derivative 17-DMAG)^[82]^. Expression and aggregation of another type Ⅲ IF, GFAP, may be reduced by phenytoin or carbamazepine. Therefore, these drugs have a potential therapeutic role in the clinical management of Alexander's disease, which is associated with heterozygous mutations in GFAP^[83]^. The major type Ⅲ IF, vimentin, is a potential molecular target for cancer therapy^[84]^. For example, the small molecule FiVe1 (https://pubchem.ncbi.nlm.nih.gov/compound/20922966) targets vimentin to promote its disorganization and phosphorylation during metaphase, leading to mitotic catastrophe and the loss of stemness. Therefore, FiVe1 has the potential to target a broad range of mesenchymal cancers^[85]^. Since cell surface vimentin is also involved in host cell interactions with pathogens, targeting such vimentin with antibodies or chemical agents that can modulate these interactions may potentially interfere with microbial pathogenesis^[86]^. For example, withaferin A, a compound derived from *Withania somnifera*, forms a direct covalent bond with vimentin by binding to Cys328 in the coil 2B domain, leading to the aggregation of vimentin filaments^[87–88]^. Similarly, withaferin A may covalently bind to human GFAP at Cys294, resulting in changes to its conformation, stability, and assembly. Following this covalent binding, withaferin A triggers the downregulation of GFAP expression at the transcriptional or post-transcriptional level, although the exact mechanism of this downregulation remains unclear^[89]^. Given the high degree of similarity between the coil 2B region of desmin and that of vimentin, it has been proposed that withaferin A may also covalently bind to desmin^[90]^. Additionally, withaferin A inhibits the phosphorylation of vimentin at Ser56^[91]^. Since vimentin and GFAP are overexpressed during gliosis, drugs targeting these proteins may have some therapeutic potential for gliosis-dependent central nervous system traumatic injury^[89–92]^. For example, withaferin A exhibits antiangiogenic and antitumor properties, along with various other biological activities, likely attributable to its interaction with diverse cellular targets. Ajoene, a phytochemical found in garlic, also covalently binds to vimentin at the same cysteine residue as withaferin A, disrupting the cellular vimentin network and reducing cell migration^[93]^. Furthermore, other natural products have been identified for their interactions with vimentin as well. Statins, such as simvastatin and mevastatin, promote the bundling of vimentin and exhibit selective cytotoxicity toward mesenchymal breast cancer cells expressing vimentin^[94]^. ALD-R491 regulates vimentin filament stability and solubility, affecting cell contractile force, cell migration speed, and directionality^[95]^. Lastly, the vimentin-targeted radiopeptide ^99m^Tc-HYNIC-(tricine/EDDA)-VNTANST shows some promise as a tool for imaging pulmonary fibrosis^[96]^.

Shikonin, a natural naphthoquinone compound derived from the roots of *Lithospermum erythrorhizon* and known for its anti-inflammatory, anticancer, and antioxidant properties, targets nestin to inhibit the hypoxia-induced proliferation of pulmonary artery smooth muscle cells^[97]^. Natural products, such as salvianolic acids, tetramethylpyrazine, and resveratrol, may induce nestin expression, although the precise mechanisms are not fully understood. The modulation of nestin through the transcriptional region by lncRNA *ENST00000448869.1* influences the pharmacological effects of chidamide in breast cancer cells^[98]^. To date, there has been no development of drugs that specifically target other type Ⅳ IFs.

Pharmacological disruption of the binding between progerin and lamin A/C using JH4, a small molecule that reduces the toxic effects of progerin accumulation and improves nuclear structure, has a beneficial effect on alleviating the symptoms of Hutchinson-Gilford progeria syndrome (HGPS)^[99]^. An improved progerin inhibitor known as SLC-D011, also referred to as progerinin (https://pubchem.ncbi.nlm.nih.gov/substance/440089748), has been shown to reduce progerin expression and improve age-related phenotypes in model systems^[100–101]^. Farnesyltransferase inhibitors are capable of interfering with abnormal splicing of the *LMNA* gene, which leads to the accumulation of progerin^[102]^. Additionally, the activation of AMP-activated protein kinase by metformin, resveratrol, or berberine may also reduce progerin production and accumulation by mitigating aberrant splicing^[103]^. Furthermore, the induction of autophagy through various means, such as rapamycin, retinoids, proteasome inhibition, and sulforaphane, facilitates the clearance of progerin, resulting in the reversal of aging-related defects in skin fibroblasts from HGPS patients and animal models^[104–105]^. Although not directly targeting lamins, the inhibition of MEK1/2, JNK, and p38α has been shown to alleviate symptoms caused by *LMNA* mutations^[106]^.

Rosmarinic acid may significantly ameliorate cataract formation and oxidative damage in the lens and increase the protein expression of filensin^[107]^. However, how rosmarinic acid acts on type Ⅵ IFs remains unknown.

## Mechanics and mechanotransduction of IFs

As cells invade the surrounding tissues, they often undergo significant deformations. While the structural integrity of eukaryotic cells under minor deformations relies on actin filaments, MTs, and IFs, it is the IF networks that play a dominant role in cytoplasmic mechanics and sustain cell viability under substantial deformations^[17,19,108–110]^. The mechanical characteristics of IFs (persistence length 0.2 to 2 μm, no loss of elasticity even at an 80% strain) differ considerably from those of actin filaments (persistence length 3 to 18 μm, loss of elasticity at approximately 20% strain) or MTs (persistence length > 1 mm, loss of elasticity at approximately 50% strain)^[108,111–113]^. Therefore, single IFs are highly flexible, and nuclear and cytoplasmic IFs also exhibit remarkable stretchability^[114]^. Depending on the experimental conditions, IFs may be stretched up to 3.6 times their original length before reaching a breaking point^[110,112]^. In addition to their high elasticity, IFs display a strain-stiffening response. IF proteins possess the unique ability to undergo molecular structural changes in response to external forces^[115]^. The initial elasticity observed at low strains largely results from the stretching of the coiled-coil α-helical domains within the IF proteins^[116]^. Further extension of the α-helical domains induces additional conformational changes, eventually leading to the formation of β-sheet structures. Consistent with this *in vitro* observation, a cysteine-reactive fluorescent probe revealed conformational changes in vimentin in cells treated with a myosin inhibitor^[117]^, and Raman microscopy provided some visual evidence that the secondary structure of vimentin changes within cells^[118]^. The conformational changes of vimentin reflect the filaments' ability to absorb energy within the mechanical range (up to 500 pN) where most physiological processes occur. Rapid stretching may result in filament stiffening at approximately 50% strain, whereas at lower velocities, IFs do not exhibit stiffness until they are stretched to approximately 200%^[116]^. The mechanical characteristics of IF networks encompass both the inherent mechanics of single molecules and the crosslinking between individual IF molecules. For example, experiments in single-cell nanomechanics have illustrated that the removal of type Ⅰ or type Ⅱ keratins leads to a reduction of over 50% in Young's modulus of keratinocytes^[119–120]^. In contrast, the overexpression of desmin or vimentin results in an increase in cell stiffness^[121]^. Additionally, vimentin IFs have been shown to play a critical role in determining cell resilience^[114]^ and in safeguarding cells from nuclear rupture and DNA damage during cell migration^[122]^.

Both keratin and vimentin play roles in mechanosensing^[123–124]^. For instance, when keratinocytes encounter varying levels of matrix stiffness, they respond by establishing a robust network of keratin bundles. This network is less susceptible to deformation, resulting in increased cell stiffness. In contrast, cells lacking vimentin exhibit impaired spreading on viscous substrates created using hydrogels with controlled elastic and viscoelastic characteristics^[124]^. More recently, vimentin IFs have been shown to modulate cellular stress by facilitating actomyosin-based force transmission and reinforcing MT networks under compression^[125]^.

**Figure 1:**
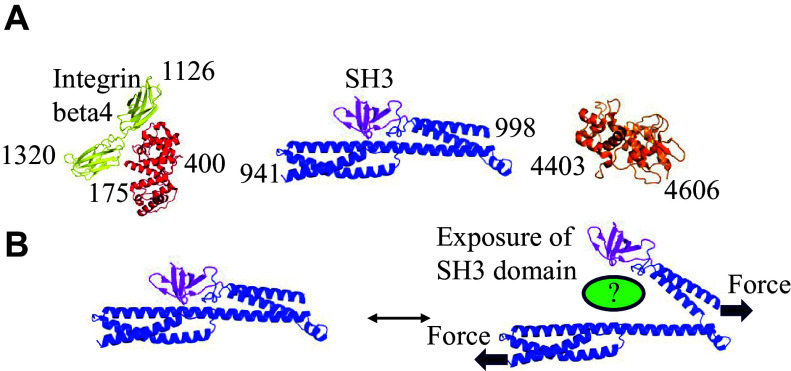
Mechanotransduction mediated by conformational changes of the plakin domain of plectin. A: The molecular structure of plectin domains. B: A model of how mechanical force exposes the SH3 domain in the plakin domain of *Caenorhabditis elegans* VAB-10/plectin. It is hypothesized that this exposure recruits an as-yet unidentified signaling molecule to transmit a signal in response to mechanical stimulation^[127]^.

IFAPs may also mediate mechanotransduction. For example, the plakin domain of *Caenorhabditis elegans* VAB-10/plectin functions as a hub in the mechanotransduction pathway that promotes morphogenesis^[126–127]^. Additionally, the loss of plectin decreases fibroblast stiffness and disrupts force transmission^[128]^. In keratinocytes, plectin also regulates nuclear mechanotransduction^[129]^ (***Fig. 1***). Although it is likely that more players are involved in mechanotransduction, the lack of structural and biochemical analysis regarding the interaction of IF with IFAPs hampers progress in this field. However, identifying additional cryptic binding sites that may be activated by mechanical forces is a challenging endeavor.

Although growing evidence indicates that IFs and IFAPs play a role in mechanotransduction, and their folded structures suggest that mechanical forces may induce unfolding and trigger biochemical signaling, this field remains in its early stages. Potential directions for future research are outlined in the final section of this review.

## Mechanics of the nucleus and mechanotransduction

Both lamin A and lamin B1 contribute to nuclear elasticity, although lamin A primarily determines nuclear viscosity^[130]^. Lamin filaments exhibit the ability to undergo reversible deformation within a low-force range (less than 500 pN), effectively serving as shock absorbers. Additionally, these filaments may endure sustained forces of up to 2 nN. These characteristics are essential for preventing filament breakage and network failure^[131]^.

Lamins transmit external and internal mechanical information to the nucleus through the cytoskeleton and the linkers of nucleoskeleton and cytoskeleton (LINC) complex (***Supplementary Fig. 4***, available online). The transmitted forces alter the nuclear structure, chromatin organization, and gene expression^[132]^.

## Conclusions and perspectives

IFs play a vital role in various fundamental biological processes. To systematically organize the genes associated with IFs and IFAPs, we have compiled a comprehensive list of all known IFs (71 genes) and IFAPs (307 genes), excluding splice variants. In this review, we have refrained from conducting an in-depth evaluation of each IFAP, as many comprehensive articles reviewing these proteins are already available, as indicated in ***Supplementary Table 2*** (available online). However, it is worth noting that the structural and biological functions of numerous IFAPs remain to be further characterized. For example, only approximately 43% (131 out of 307) of the known IFAPs have been demonstrated to directly interact with IFs *in vitro*. Future research endeavors are necessary to validate the direct interactions of the remaining 174 IFAPs with IFs and to delve into more intricate details of these interactions. Some IFAPs lack well-defined IF interaction domains, and it is possible that only specific splice variants engage with IFs. Identifying and characterizing IFAPs remains a labor-intensive task that demands extensive biochemical work in wet laboratories. Currently, computational algorithms for discovering new IFAPs using IF-binding motifs are not available. Despite these challenges, more IFAPs are likely to be discovered in the future, particularly those enzymes involved in the post-translational modifications of IFs. Such structural and biochemical characterizations are crucial for unraveling the molecular mechanisms of mechanotransduction.

Considering that IFs and IFAPs may experience mechanical stress from both external and internal forces, it is conceivable that such stress may expose previously unrecognized binding domains that have not been considered drug targets before. Because of their fundamental roles in normal cells, the specific manipulation of IF systems for therapeutic purposes presents a formidable challenge. Nonetheless, some research groups have initiated screenings for drugs that modulate IFs and IFAPs^[85,133–134]^. To facilitate these efforts, it is essential to gain a deeper understanding of the molecular mechanisms that govern IF dynamics, mechanotransduction, and their interactions with IFAPs. This involves exploring how mechanical forces induce changes in protein conformation, potentially revealing new druggable sites. Moreover, the development of more advanced screening techniques, such as high-throughput platforms or structure-based drug design, will be crucial for identifying compounds that may specifically target these newly exposed binding domains without affecting normal cellular functions. Such advancements may pave the way for the precise therapeutic targeting of IFs and IFAPs in various disease contexts.

Finally, drawing on our previous research experience with mechanotransduction mediated by the actin cytoskeleton and the functions of IFAPs summarized in ***Supplementary Table 2***, as well as the mechanical and structural properties of IFs described above, we have proposed our hypotheses and remaining issues regarding mechanotransduction involving IFs.

1. Since IFs may be stretched up to 3.6-fold before reaching a breaking point, significant structural changes likely occur in IF molecules under mechanical stress. These structural changes would regulate the interaction with IFAPs, but the following issues need clarification: (a) The stretching of IFs induces conformational changes in the coiled-coil α-helical domains but likely has little influence on the structure of the head and tail domains of IFs (***Supplementary Fig. 2***, available online). As summarized in ***Supplementary Table 2***, only a limited number of IFAPs have been thoroughly characterized regarding the specific IF domains that they bind to. Identifying these binding domains is essential to understanding how mechanical forces may influence IF-IFAP interactions. Furthermore, the current molecular structures of IF-IFAP complexes do not clearly suggest a role in mechanotransduction. Although AlphaFold predictions may offer valuable insights into these complex structures, a high-throughput screening approach is still needed to identify the specific mechanosensing domains. (b) The biophysical properties of IFs have been characterized using purified proteins, but it remains unclear how IFs deform under physiological forces. Developing a quantitative force sensor is essential (for instance, as described in a reference^[135]^), but designing such a sensor requires identifying the mechano-sensing domain and understanding its structure.

2. Mechanosensitive transcription factors or cofactors, such as YAP1, shuttle between the nucleus and cytosol, with this translocation regulated by the actin cytoskeleton^[136–137]^. Similarly, the translocation of HNRNPK from the nucleus to the cytoplasm promotes cell proliferation and cancer metastasis. KRT19 directly interacts with HNRNPK and sequesters it in the cytoplasm. In the absence of KRT19, HNRNPK localizes to the nucleus, resulting in a reduced cell proliferation^[138]^.

As summarized in ***Supplementary Table 2***, several IFAPs, such as HNRNPC, CRHBP, HNRNPA1, PPARG, and RNF26, are also localized in the nucleus despite their interactions with vimentin. Therefore, it is possible that the nucleocytoplasmic shuttling of these proteins is regulated by mechanical forces, as these forces may influence the interaction between IFAPs and IFs, as discussed above.

3. When mechanical forces are applied to tissues, they become stronger to withstand them. For instance, calluses contain keratin, which reinforces and protects the skin against mechanical stress^[139]^. This suggests that mechanical stress triggers the gene expression of IFs and IFAPs. However, the exact mechanotransduction pathways remain to be discovered, requiring future research that may ultimately aid in designing small-molecule inhibitors.

## SUPPLEMENTARY DATA

Supplementary data to this article can be found online.
