# Supplementary material for: Intermediate filaments and their associated molecules
Source: J Biomed Res. 2025 Feb 8;39(3):242–53. doi: 10.7555/JBR.38.20240193 (PMC12239979; doi:10.7555/JBR.38.20240193)
Supplement: Supplementary file 1 — Supplementary data to this article can be found online. [file jbr-39-3-242-Supplementary.pdf]

Available online at www.jbr-pub.org.cn

Open Access at PubMed Central

Journal of Biomedical Research, 2025 39(3): 1–20

*Supplementary Data*

JBR

# Intermediate filaments and their associated molecules

Jing Gao, Fumihiko Nakamura✉

*School of Pharmaceutical Science and Technology, Tianjin University, Tianjin 300072, China.*

## IF gene families and their protein structures

Human IF proteins evolve from a common lamin-like ancestor, are encoded by one of the largest human gene families (73 genes), and subcategorized into six types (***Supplementary Table 1***, available online)[1–4]. Expression of IFs is highly regulated during different stages of development and differentiation, and some of the IF genes give rise to multiple splice variants[5–6] (see link to Uniprot in ***Supplementary Table 1***).

Type Ⅰ (28 genes) and type Ⅱ (27 genes) families encoded on either chromosome 12 or 17 encode acidic (isoelectric point (pI) = 4.4–6.1, calculated by https://web.expasy.org/compute_pi/) and basic (pI = 5.4–8.4) keratins (KRTs), respectively. Although the overall pIs of the basic keratins are not basic, their N- and/or C-terminal regions are basic. Amino acid sequence identity and similarity of type Ⅰ and type Ⅱ IFs calculated by the SIAS tool (http://imed.med.ucm.es/Tools/sias.html) were as follows: type Ⅰ, identity = 4.1%–22%, similarity = 10%–28%; and type Ⅱ, identity = 4.1%–39%, similarity = 7.8%–46%). Notably, KRT6A–C have a high identity within them (identity > 98%) and are also similar to KRT84 (similarity approximately 45%). Human keratins are all α-keratin, which is usually found in soft tissues, such as skin, nails, and hair, while β-keratin is found in hard tissues, such as horns, claws, and bird feathers[7]. Although keratins are highly expressed in epithelia, they are also expressed in non-epithelial cells[8].

Type Ⅲ (five genes) encodes vimentin, desmin, syncoilin, peripherin (neurofilament 4), and glial fibrillary acidic protein (GFAP), with sequence identity ranging from 6% to 11% and similarity 12% to 17%. Although some researchers have categorized peripherin as a type Ⅲ IF[2,4,9–10], others have demonstrated that peripherin is part of the neurofilaments (NFs), which are categorized in type Ⅳ IF[11–12]. Type Ⅲ IFs are important components of the cytoplasmic cytoskeleton and are expressed in specific cell types. Vimentin, the ubiquitously expressed and well-characterized type Ⅲ IF protein, is mainly expressed in cells of mesenchymal origin[13]. Desmin and syncoilin are highly expressed in skeletal and cardiac muscle, and syncoilin binds to desmin and α-dystrobrevin in the sarcomere Z-disk[14–15]. GFAP is mainly expressed in glial cells[16].

Type Ⅳ (six genes) encodes three neurofilament (NF) polypeptides (*i.e.*, light, medium, and heavy chains), α-internexin, nestin, and synemin, with sequence identity ranging from 7% to 13% and similarity 13% to 19%. NF polypeptides are the major IFs of many types of mature neurons and are abundant in the axons of long motor neurons[17]. Before the expression of the NF polypeptides, α-internexin is expressed at an earlier stage of the development of neurons[18]. α-Internexin appears to be a bona fide 4th NF subunit and is incorporated into the three NFs in the central nervous system[18]. Peripherin is also incorporated into the triplet NFs in the peripheral nervous system[12]. Therefore, NFs are composed of four IFs. Nestin (neuroepithelial stem cell protein) is expressed in mitotically active cells. Moreover, its expression level changes during development and is replaced with other IFs in some tissues[19]. Synemin,

✉Corresponding author: Fumihiko Nakamura, School of Pharmaceutical Science and Technology, Tianjin University, 92 Weijin Road, Nankai District, Tianjin 300072, China. E-mail: fnakamura@tju.edu.cn.

Received: 03 July 2024; Revised: 23 November 2024; Accepted: 25 November 2024; Published online: 08 February 2025

CLC number: R329.28, Document code: A

© 2025 by Journal of Biomedical Research.

The authors reported no conflict of interests.

This is an open access article under the Creative Commons Attribution (CC BY 4.0) license, which permits others to distribute, remix, adapt and build upon this work, for commercial use, provided the original work is properly cited.

https://doi.org/10.7555/JBR.38.20240193

also known as desmuslin, is mainly expressed in muscle cells and various other cell types[20]. Synemin proteins do not autonomously form filaments but co-polymerize with vimentin and desmin[21].

Type Ⅴ (three genes) encodes nuclear lamins, characterized by 6%–10% sequence identity and 13%–17% similarity. Unlike other IFs, the nuclear lamins are components of the nuclear envelope (NE). Previous observations noted that nuclear lamins formed an orthogonal meshwork underlying the nuclear membrane[22–23], but recent research has observed that nuclear lamins form various geometries[24–25]. Lamins A and C (A-type) are generated from the *LMNA* gene by alternative splicing[26]. Their first 566 amino acids are identical, but their carboxyl-terminal domains differ. Unlike lamin C, prelamin A is processed to mature lamin A by farnesylation, carboxyl methylation, and endoproteolytic cleavage[27]. Lamin B1 and B2 (B-type) are independently produced from *LMNB1* and *LMNB2* genes, respectively[28].

Type Ⅵ (two genes) encodes filensin, phakinin, and beaded filament structural protein 1 and 2 (BFSP1 and BFSP2), with sequence identity approximately 9.6% and similarity approximately 15.4%, respectively. Beaded filaments manifest as unique beaded structures in lens fiber cells, distinguishing them structurally and functionally from the conventional IFs observed in other cell types. Although they share certain structural traits with IFs, such as the capacity to form filamentous structures, their specialization lies in fulfilling the transparency and refractive demands specific to the lens. Consequently, type Ⅵ IFs are typically categorized as an "orphan" subgroup within the IF protein family[29]. However, due to the ability of filensin and phakinin proteins to form 10-nm filaments *in vitro* and their primary sequence similarity to other IF family members, we have included them as constituents of the IF protein family in this review.

## Protein structures and assembly mechanisms of IFs

The primary structure of IFs indicates that the central rod domains of all IFs are relatively consistent in size (300–350 aa), whereas N-terminal head and C-terminal tail domains vary (see number of amino acid residues shown in ***Supplementary Table 1***). For example, type Ⅳ IFs (the major IFs of many types of neurons) have relatively long tails, and type Ⅴ IFs (lamins) have an immunoglobulin-(Ig)-like domain in the tails[4]. Lamin and vimentin are the best-characterized IFs so far, which proposed a common assembly mechanism of IFs (***Supplementary Figs. 1*** and ***2***), although some distinctive assembly mechanisms were proposed in vimentin, lamin, and keratin[30–32].

The core rod domain is composed of an α-helical coiled-coil (CC) structure, which is originally divided into four helical domains (1A, 1B, 2A, and 2B) separated by linkers (L1, L12, and L2) (***Supplementary Fig. 1***)[33–34]. Structural analysis of the coil domain revealed that the entire region of coil 2 forms a continuous CC structure[35–36], leading to renaming the subdomains to coils 1a, 1b, and 2, while the same is true for the coil 1 region (L1 also forms a continuous CC structure). However, the classical nomenclature is still useful because domains 2A and 2B have distinctive functions during the assembly of IF and are thus implicated in disease phenotypes. For example, the majority of the mutations reported to date lie in the 2B region (hot spot) because it plays a critical role in the overlapping interactions during IF assembly (***Supplementary Fig. 2***)[37].

The formation of a parallel dimer is believed to be the first step of IF assembly (***Supplementary Fig. 2***). Type Ⅰ and type Ⅱ keratin assemble into a parallel homo- or hetero-dimer, and others form parallel homodimers, although desmin and vimentin also form a heterodimer[38–40]. While the exact arrangement of the dimers within IFs is difficult to experimentally determine[41], some theoretical models have been suggested based on structural analysis of the fragments of IF molecules and chemical cross-linking experiments. The two dimers form an anti-parallel tetramer, and eight tetramers assemble into a unit-length filament (ULF)/protofibril. Then the ULF assembly into an apolar rope-like IF filament, which contains four different binding modes of the dimers, *i.e.*, $A_{11}$, $A_{22}$, $A_{12}$, and $A_{CN}$[42–44]. As the names indicate, $A_{11}$, $A_{22}$, $A_{12}$, and $A_{CN}$ are mediated by interactions of 1B domain, 2B domain, both domains 1 and 2, and tail (C-terminal)-to-head (N-terminal), respectively (***Supplementary Fig. 2***).

The assembly mechanisms and detailed structures of keratins differ from vimentin or lamin. Keratins are highly enriched in low-complexity aromatic rich kinked segments (LARKS) in the head and tail domains. Since LARKS mediates the stacking of segments into kinked β-sheets that pair into protofilaments[45] and keratin-associated proteins also contain LARKS, LARKS appears to play an important role in keratin assembly. On the other hand, for IFs that lack or have fewer LARKS, such as vimentin and lamins, $A_{CN}$ is connected by overlapping the rod

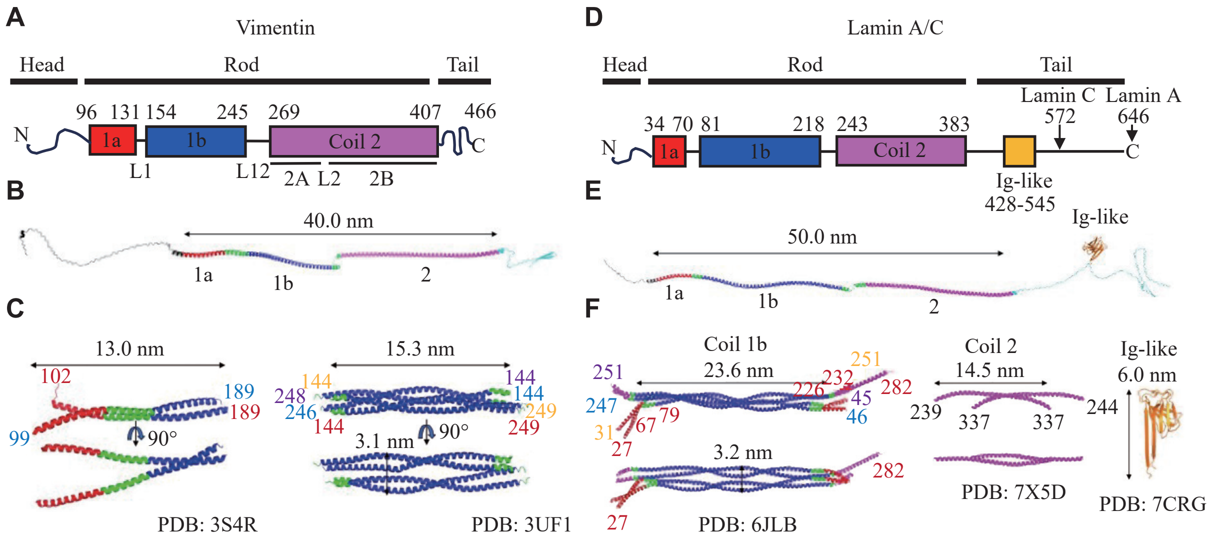


***Supplementary Fig. 1*** **Structures of intermediate filaments.** A and D: Schematic depictions of the structures of human vimentin and lamin A/C. The box indicates coil domains. L1, L12, and L2 indicate the linker. The coil 2 segment is further divided into coil 2A and 2B, which are connected by the linker L2. B and E: Full-length structures of human vimentin and lamin A/C predicted by AlphaFold (https://alphafold.ebi.ac.uk). N-terminal unstructured head (black), coil 1a (red), linkers (green), coil 1b (blue), coil 2 (magenta), C-terminal unstructured tail (cyan), and Ig-like domain of lamin A/C (orange). The predicted structures are extended for display purposes. C and F: Crystal structures of coil domains of human vimentin and lamin A/C. The structure of the Ig-like domain of human lamin A/C is shown in F.

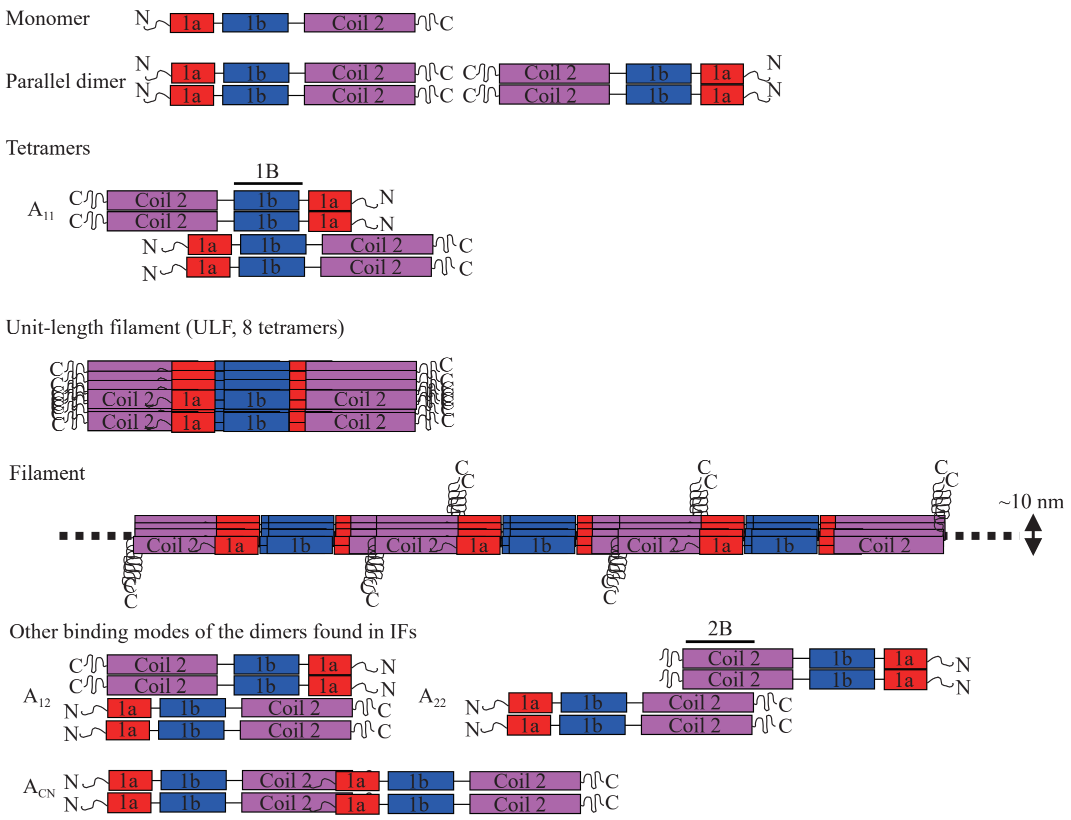


***Supplementary Fig. 2*** **Assembly mechanism of intermediate filaments (IFs).** Monomers assemble into parallel dimers. The two dimers form an anti-parallel tetramer, eight tetramers assemble into a unit-length filament (ULF)/protofibril, and then ULFs assemble into the rope-like IF filament bundle. Four different binding modes of the dimers have been identified in the complete IFs. $A_{11}$, overlapping of antiparallel 1B segments; $A_{22}$, overlapping of antiparallel 2B segments; $A_{12}$, overlapping of antiparallel 1B and 2B segments; and ACN, short head-to-tail overlapping between parallel molecules. The head and tail domains are likely free from the fiber and susceptible to post-translational modifications. The formation of tetramers and ULFs is regulated by the phosphorylation of residues in the head domain of IF proteins.

domain ends[46–47]. Unlike microtubules (MTs) and actin filaments, the IF assembly process does not require cofactors and nucleotides[2]. In these IFs, the head and tail domains are likely free from the fiber

and susceptible to post-translational modifications (PTMs). Nevertheless, the disordered head domains might play a role in facilitating the assembly of IFs[48]. In KRT5 and KRT14, the crystal structure of the interacting 2B regions within their coiled-coil domains reveals an asymmetric interface characterized by salt bridges, hydrogen bonds, hydrophobic interactions, and surface charge polarization. A trans-dimer disulfide bond involving KRT14-Cys367 is present in the crystal and influences nuclear shape, with keratinocytes lacking KRT14 showing abnormalities in nuclear structure[49]. Additionally, the crystal structure of human KRT1/KRT10 helix 1B heterotetramer reveals a knob-pocket mechanism in keratin assembly[4,50]. Moreover, a recent study using cryo-focused ion-beam milling and cryo-electron microscopy has provided a detailed *in situ* structure of vimentin IFs[51]. *In situ*, vimentin IFs are made up of five protofibrils, with intrinsically disordered head domains forming a fiber within the lumen and tail domains establishing lateral connections between the protofibrils. This technique offers great promise for revealing the structure of other IFs *in situ* as well.

## Expression, PTMs, and turnover of IFs

### Keratin IFs (type Ⅰ–Ⅱ)

Human keratins have highly conserved rod domains, yet they are uniquely expressed in different tissues with homodimeric or distinctive heterodimeric pairs (*e.g.*, KRT1/KRT10, KRT5/KRT14, KRT6/KRT16, KRT7/KRT19, and KRT8/KRT18), termed interaction pairs at different stages of development, differentiation, and disease development[52–54]. The programmed death of the keratinocytes occurs during keratinization (cornification) in hair, skin, and nails, which is different from apoptosis[55]. The densely packed keratins, keratin-associated proteins (KRTAPs), and other proteins in dead cells form a rigid structure through disulfide bond cross-linking. By contrast, the turnover of keratins in living cells is highly dynamic, but little is known about the mechanism of their degradation. Nevertheless, the ubiquitin-proteasome system is believed to be an important regulator[56]. Besides ubiquitination, keratins are also subject to phosphorylation, glycosylation, SUMOylation, acetylation, and transamidation[57] (***Supplementary Fig. 3***). Although the biological functions of these PTMs are not fully understood, phosphorylation is known to enhance keratin solubility and increase resistance to apoptosis. In human KRT8, Ser-23 and Ser-431 in the head and tail, respectively, are the major phosphorylation sites[58]. Ser-23 is highly conserved among all type Ⅱ keratins, and phosphorylation at Ser-431 occurs after epidermal growth factor stimulation and during mitotic arrest. However, other research showed that KRT8 Ser-73 phosphorylation might serve as a marker for stress, mitosis, and apoptosis in simple epithelia[59]. Phosphorylation of KRT8 has also been shown to regulate epithelial-mesenchymal transition and cell migration[60–61]. Phosphorylation of KRT8 at Ser-43 by mechanical overloading activates Ras homolog family member A, which in turn activates protein kinase N, leading to the suppression of autophagosome initiation and contributing to mechano-stress-induced intervertebral disc degeneration[62]. In KRT19, Try-391 in the tail domain is phosphorylated in the presence of Src kinase *in vitro* and in cells expressing constitutively-active Src[63]. Interestingly, although vimentin does not considerably copolymerize with keratin, keratinocyte migration requires vimentin-keratin interactions[64].

The observation of the asymmetric distribution of specific mRNAs with cytokeratin within the *Xenopus* egg, along with the interaction of mRNA with vimentin, suggests that RNA-IF interactions may play a role in the localization of protein expression and stabilization of mRNA[65–67]. It has been found that KRT19 directly interacts with heterogeneous nuclear ribonucleoprotein K (HNRNPK), a regulator of pre-mRNA processing and long non-coding RNA localization in the nucleus[68]. This interaction sequesters HNRNPK in the cytoplasm. The loss of KRT19 results in the loss of cytoplasmic localization of HNRNPK and reduces cell proliferation[68]. Other non-lamin IF proteins also play significant roles in the nucleus in various cell types[69].

### Type Ⅲ

Type Ⅲ IF proteins form homodimers and heterodimers with other type Ⅲ and some type Ⅳ IFs, such as nestin, neurofilaments, and synemins[13]. Each type Ⅲ IF protein is highly dynamic and involved in many cellular functions and diseases as follows.

*Vimentin*

Vimentin is abundantly expressed in cells of mesenchymal origin, thereby serving as a marker of mesenchymally-derived cells or cells undergoing an epithelial-to-mesenchymal transition[70–71]. Vimentin is highly modified as listed in PhosphoSite v6.6.0.4 (www.phosphosite.org)[72] (***Supplementary Fig. 3***), and phosphorylation is the key regulator of vimentin assembly and disassembly[73–75], with the N-terminal

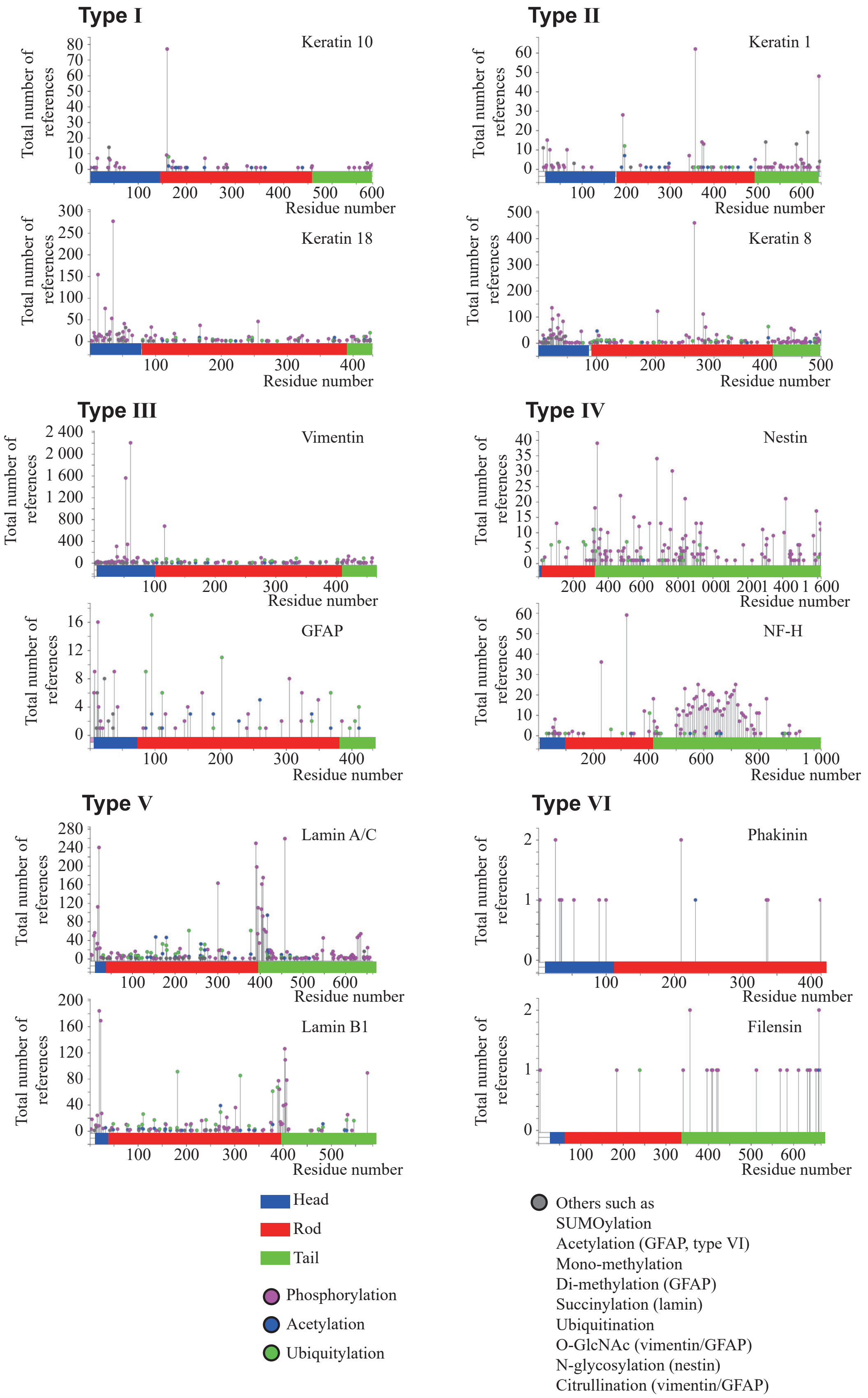


***Supplementary Fig. 3*** **Post-translational modifications of intermediate filament (IF) proteins in different types.** It should be noted that the size of full-length IF proteins varies. The red bar indicates the central rod domains that are relatively uniform in size (300–350 aa) and assemble into a parallel heterodimer, whereas N-terminal head and C-terminal tail domains vary in size. The graphs were generated on the PhosphoSitePlus website (https://www.phosphosite.org/homeAction).

head domain phosphorylated by multiple kinases. For example, protein kinase A (PKA) phosphorylates Ser38/72 to decelerate the assembly kinetics of vimentin subunits[73]. Src-related kinase lacking C-terminal regulatory tyrosine and N-terminal myristoylation sites, also known as protein tyrosine kinase 70 (PTK 70), phosphorylates vimentin at Tyr61 and Tyr383[76]. Moreover, in human prostate cancer cells, atypical protein kinase Cs (PKCs) have been found to phosphorylate vimentin at Ser33/39/56 at the cell front, thereby promoting local disassembly of vimentin and enhancing cell motility and invasion[77]. Phosphorylation at Ser56 is also mediated by the cyclin-dependent kinase 1 and Rho-associated protein kinase. In the C-terminal tail domain, polo-like kinase 1 phosphorylates Ser459 to inhibit endocytic vesicle fusion in mitosis[78]. Other modifications, such as SUMOylation, glycosylation, acetylation, and citrullination, are implicated in vimentin solubility, assembly, and cell migration, respectively[79–80]. Ubiquitination of vimentin is regulated by gigaxonin, which interacts with vimentin and cullin 3, a component of E3 ubiquitin ligase complexes, and promotes the proteasomal degradation of soluble vimentin[81]. Gigaxonin deficiency leads to the accumulation of not only vimentin but also other IF proteins in neuronal axons, resulting in giant axonal neuropathy[82]. It has been proposed that accumulated soluble vimentin sequesters an unidentified adaptor that links kinesin to vimentin, thereby disrupting the interaction between vimentin and the kinesin-1 motor and impeding vimentin transport along MTs[81]. Citrullinated vimentin plays a role as an autoantigen in the development of rheumatoid arthritis and contributes to antitumor immune responses[83]. GFAP is also citrullinated and detected in several diseases, such as Alzheimer's disease and retinal injury[84–85].

Vimentin interacts with MTs and actin filaments to regulate its subcellular organization and transport[86–88]. Vimentin also stabilizes MTs by direct interaction[89]. Vimentin binds to actin filaments both directly and indirectly, forming an interpenetrating network that contributes to cell mechanics[90]. Vimentin-deficient mice have exhibited defects in wound healing, vascular constriction or tone, fibrosis, cell metastasis, inflammation, fat metabolism, neurogenesis, and mechanotransduction[91]. In some pathological conditions like inflammation, vimentin filaments depolymerize because of phosphorylation or citrullination, a process in which the arginine amino acid is converted into citrulline amino acid. Subsequently, vimentin oligomers are released into the extracellular space, where they interact with the extracellular matrix (ECM) and cells to mediate cell adhesion and pathogen-host interactions[92–94]. Vimentin also binds to G-quadruplex DNA at telomeres and gene promoters to regulate gene expression during cell development and migration[95].

### *Desmin*

Desmin is a major IF in striated and smooth muscle cells[96–98]. It localizes at the Z-disks to wrap the myofibrils and is connected to the nuclear envelope, the costameres of the sarcolemma, and other cellular organelles, such as mitochondria, T-tubules, and the sarcoplasmic reticulum. Desmin also anchors the myofibrils to the ECM through the plasma membrane. These connections are mediated by various adaptor proteins. Nevertheless, desmin null mice are viable and fertile, indicating that desmin is not essential for myogenesis, presumably because the loss of desmin elicits compensatory responses to stabilize the myofibrils, although some morphological abnormalities are detected in muscle tissues[99–100]. In humans, mutations in desmin often cause cardiomyopathies and skeletal muscle atrophy[101–102]. Similar to other IFs, phosphorylation in the head domain by various kinases promotes the solubilization of desmin IF[97]. In atrophying muscles, phosphorylation of desmin leads to its ubiquitination and promotes its cleavage by the $Ca^{2+}$-dependent protease calpain-1[103]. Furthermore, in myofibrillar myopathy, mutations in desmin and hyperphosphorylation by PKCzeta trigger the aggregation of desmin, which subsequently leads to the misfolding of native desmin protein[104–106]. Notably, such aggregation has been slowed down by the green tea polyphenol epigallocatechin gallate[106]. Besides mechanical support for structural integrity, desmin appears to play roles in metabolism and signal transduction[97].

Additionally, desmin IFs influence the movement and orientation of actin filaments, slowing their speed upon contact and broadening the difference between incoming and outgoing angles. Desmin IFs also bind to heavy meromyosins and weaken actin-myosin interactions, potentially affecting the dynamic organization of cell architecture[107].

### *Syncoilin*

Syncoilin is highly expressed in striated muscle but not in smooth muscle cells[15,108–109]. Syncoilin is also expressed in both the central and peripheral nervous systems, with different isoforms being expressed in different neurons (*e.g.*, isoform 1 in the brain and isoform 2 in the spinal cord and sciatic nerve)[110].

Syncoilin binds alpha-dystrobrevin and desmin to link muscle IFs to ECM through the dystrophin-associated protein complex. Similar to desmin, syncoilin is dispensable for cardiac and skeletal muscle development but is necessary for efficient lateral force transmission during contraction[111].

*Peripherin*

Peripherin shares more than 70% sequence homology with other type Ⅲ IF proteins but is predominantly expressed in neurons in the peripheral nervous system[112]. Although peripherin null mice do not show apparent phenotypes with normal development and reproduction[113], loss of peripherin results in the reduction of L5 unmyelinated sensory fibers[113] and noise-induced hearing loss[9,114]. In humans, peripherin is involved in several diseases, including amyotrophic lateral sclerosis (ALS), a neurodegenerative condition affecting nerve cells in the brain and spinal cord, leading to loss of muscle control; Hirschsprung disease, a congenital condition characterized by missing nerve cells in the colon, causing severe constipation or intestinal blockage; and other diseases such as autoimmune and infectious conditions[9]. For instance, aberrant expression, mutation, and modification of peripherin are observed in ALS patients, although the disease mechanisms remain poorly understood. Additionally, peripherin interacts with the small GTPase RAB7A, which is mutated in the neurodegenerative disease Charcot-Marie-Tooth type 2B, characterized by progressive muscle weakness and atrophy. Since disease-causing RAB7A mutant proteins interact more strongly with peripherin and promote solubilization of peripherin, pharmacological perturbation of this interaction is a potential therapeutic target[115].

*GFAP*

GFAP is primarily expressed in astrocytes (glial cells) and neural stem cells[116–119]. Since 12 isoforms are predicted to be expressed in humans and GFAP colocalizes with vimentin, GFAP IF networks likely contain multiple isoforms and other IFs. Similar to other IF proteins, the disassembly of GFAP is also regulated by phosphorylation of the head domain[120]. Interestingly, fluorescence recovery after photobleaching experiments demonstrated that different isoforms have distinctive dynamics in living cells, presumably because of different PTMs and structural properties[121]. Since GFAP protein levels are frequently changed in gliomas, it was expected to be used as a biomarker for diagnosis. GFAP levels in serum are more consistently associated with the malignancy grade of gliomas and are promising biomarkers for astrocytopathy[122–124]. For example, autoimmune GFAP astrocytopathy is an inflammatory central nervous system disorder and the level of anti-GFAP antibodies in serum and cerebrospinal fluid is associated with hypertrophic pachymeningitis[125]. Nonetheless, the variability among patients and inconsistent results across various studies indicate limitations in the usefulness of GFAP levels as a biomarker. Further research is necessary before the clinical application of these findings[126].

## Type Ⅳ

*Neurofilament proteins*

Neurofilament proteins are predominantly expressed in neurons and subcategorized into neurofilament light (NF-L), neurofilament middle (NF-M), and neurofilament heavy (NF-H) with different molecular weights (***Supplementary Table 1***). NF-L forms homo-oligomers *in vitro*[127], whereas *in vivo*, all available evidence suggests that NFs are heteropolymers composed of four subunits: NF-L, NF-M, NF-H, and α-internexin (INA) or peripherin[128–129]. The major difference between the three neurofilament proteins is attributed to their C-terminal tails. The tail domains of NF-M and NF-H contain multiple lysine-serine-proline (KSP) repeats that are heavily phosphorylated and project from the surface of the filament core due to the extensive negative charges[129]. These tail domains are also O-linked glycosylated[130], which competes with protein phosphorylation; thus, increased O-linked N-acetylglucosamine (O-GlcNAc) levels reduce the phosphorylation of these domains[131]. Since disease-causing mutations are found in the tail domains, especially in KSP repeats, they may have significant biological functions. However, the biological role of these PTMs is not fully understood[132]. Similar to other IFs, phosphorylation of the N-terminal head domains by various kinases, such as PKA and PKC, promotes the disassembly of NF[133]. Interestingly, phosphorylation of the NF tail domains by cyclin-dependent kinase 5 and mitogen-activated protein kinase is involved in axonal elongation[134]. NF proteins (proteolytic fragments) are detectable and measured in both the cerebrospinal fluid and blood, serving as a biomarker of neurodegenerative diseases, such as ALS and Charcot-Marie-Tooth disease, the latter being characterized by progressive muscle weakness, atrophy, and sensory loss in the peripheral nerves[135–136].

*INA*

INA is mainly expressed in central and peripheral

neurons and is frequently detected in medulloblastoma, neuroblastoma, and gastroenteropancreatic neuroendocrine neoplasms[137–140]. Therefore, INA may be used as a biomarker for these diseases as well as for ALS and other motor neuron diseases[141–143]. INA assembles similarly to other IFs *in vitro*[144], yet *in vivo*, it is integrated into the triple NFs[18], and phosphorylation of the head domain also regulates the disassembly of the filament[145]. Epigenetic inactivation of INA by hypermethylation in CpG islands located in the promoter region is frequently detected in adenocarcinoma[146]. Such hypermethylation is a prognostic marker for poor overall survival in patients with colorectal cancer. Restored INA expression blocks migration and invasion of cancer cells and reduces lung metastasis by directly inhibiting the polymerization of MTs[146].

### *Nestin*

Nestin is known as a marker of neural stem/progenitor cells but is also ubiquitously expressed in various tissues and highly expressed in some cancer cells[147–148]. The nestin protein has a long C-terminal tail, which may prevent the association of vimentin with actin filaments because nestin copolymerizes with vimentin[19,149]. Since the association of vimentin with actin filament stiffens the filament[150], nestin softens the cell body, and overexpression of nestin promotes cell migration and metastasis[149].

### *Synemin*

Synemin is mainly expressed in muscle, testis, breast, and soft tissues[20,151–153]. Humans express three isoforms (1 565 aa, 1 253 aa, and 339 aa) by alternative splicing. The two larger isoforms of synemin, *i.e.*, α- and β-synemin, have extended C-terminal tails that radiate outward from the surface of the filament and provide connecting arms that associate with other proteins, such as talin, vinculin, alpha-dystrobrevin, desmin, dystrophin, and utrophin[20,154–157]. Isoform 2 (also known as desmuslin) is strongly detected in adult heart, fetal skeletal muscles, and fetal heart, and colocalizes to the Z-lines[156]. Although synemin knock-out mice are viable and fertile, and synemin is not required for embryonic development and myogenesis, the loss of the synemin gene leads to cardiac and skeletal myopathies. In humans, synemin gene mutations have been linked to dilated cardiomyopathy and skeletal myopathy[20,156–158]. Besides providing mechanical integrity, synemin has been recently implicated in other biological functions, such as the PKA-mediated signaling pathway and DNA-damage response[20].

## Type V

The nuclear lamina is composed of lamins and nuclear lamin-associated membrane proteins, forming a meshwork lining the inner surface of the nuclear envelope[31,159–163]. Both lamin A/C and B directly bind to core histones through a specific sequence element in their tail domain[164]. The tail domain also contains a nuclear localization signal and an Ig-like domain that mediates dimerization[165] (***Supplementary Fig. 1***). The nuclear envelope lamina is reversibly depolymerized during mitosis by phosphorylation of the head domains by multiple kinases, such as cyclin-dependent kinase and PKA[159,166–167]. Mutations in the *LMNA* gene lead to defective mechanotransduction and human diseases known as laminopathies[168–171]. Examples of laminopathies include Emery-Dreifuss muscular dystrophy (EDMD), which affects skeletal and cardiac muscles, causing muscle weakness, joint contractures, and heart issues; Hutchinson-Gilford progeria syndrome (HGPS), a premature aging disorder in which children show signs of accelerated aging; lipodystrophy, a condition marked by abnormal fat distribution in the body; and dilated cardiomyopathy, where the heart becomes enlarged and weakened, impairing its ability to pump blood effectively. Mouse embryonic cells do not express A-type lamins, implying that the presence of lamin A/C may act as a constraint on the adaptability of cells for subsequent developmental processes[172]. In line with this observation, A-type lamins are non-essential in human cells, whereas B-type lamins (B1 and B2) are indispensable[173]. Nevertheless, mice deficient in both B-type lamins develop until birth but do not survive beyond immediate postnatal stages[174]. Notably, lamins are dispensable for the survival, proliferation, and differentiation of embryonic stem cells[175], again suggesting the role of nuclear lamins in the context of tissue formation and maintenance.

### *Lamin A and C (type A)*

Lamin A and C (type A) are proteins encoded by the *LMNA* gene in humans, and each mature lamin is produced by different processing mechanisms[22,176]. For lamin A, the *LMNA* gene expresses prelamin, which is prenylated at Cys661. Prenyl-CaaX-specific endoprotease removes the tripeptide at the carboxyl terminus of prenylated prelamin A, and then RAS converting enzyme 1 (RCE1) and zinc metalloproteinase Ste24 homolog (ZMPSTE24) cleavage at 646–647 to produce mature lamin A. Although pre-lamin A processing appears to be

important for heterochromatin remodeling, the role of these processing events is not fully understood[177]. Unlike lamin A, lamin C is produced without a CaaX box due to alternative splicing and is therefore not processed (***Supplementary Fig. 1***).

The expression of lamin A/C, but not lamin B, is associated with tissue stiffness and is upregulated when cells are cultured on stiff substrates or exposed to mechanical stretching[178–180]. In contrast, culturing cells on soft substrates or inhibiting myosin Ⅱ activity leads to a decrease in lamin A/C levels, likely because of increased phosphorylation and solubilization[178]. Additionally, adherent cells exhibit higher lamin A/C levels than non-adherent cells, highlighting the critical role of lamin A/C in mechanotransduction within adherent cells. *LMNA* mutations causing muscular dystrophies increase Yes Associated Protein 1 (YAP1) nuclear entry in muscle stem cells, even at high cell density[181]. HGPS is a very rare and complex syndrome because of mutations in *LMNA*[102,182–184]. In HGPS, C-terminally truncated lamin A (pre-progerin), which causes nuclear deformation at the inner nuclear membrane (INM), is expressed and farnesylated through the exposed cysteine residue to produce progerin. However, this farnesylated progerin fails to be cleaved by ZMPSTE24 metalloproteinase to produce mature lamin A[185]. Additionally, the C-terminal cysteine (Cys611) residue of progerin (LMNA isoform 6) forms a disulfide bond with the cysteine residue in the Ig-like domain of lamins, indicating that unfarnesylated progerin can establish a disulfide bond with the Ig-like domain within the lamin network[186]. Reduced expression of lamin A/C is also frequently associated with cancer phenotypes[187]. These mutations and decreased lamin A/C expression may induce genomic instability[170], potentially by disrupting the normal association of the genome with the nuclear lamina. Lamins directly bind to chromatin or indirectly through an adaptor protein such as barrier-to-autointegration factor 1 (BAF/BANF1; ***Supplementary Fig. 4***). Dysfunction of lamin A/C affects the stability of the genome, as lamina-associated domains (LADs) occupy about 30–40% of the total genome, and genes localized within LADs are typically transcriptionally silenced[188].

*Lamin B1 and B2 (type B)*

Lamin B1 and B2 (type B) are proteins encoded by distinct transcripts originating from the *LMNB1* and *LMNB2* genes[171]. Similar to lamin A, the B-type lamins are also processed with some differences. For example, only RCE1 is responsible for the cleavage of the B-type lamins, and the C-terminal farnesylcysteine is methylated by an isoprenylcysteine carboxyl methyltransferase and is permanently farnesylated and carboxymethylated. Deficiency in the endoproteolysis by RCE1 or methylation of lamin B1 results in loss of integrity and deformity of the nuclear lamina[189]. However, another group reported that the stability of the lamin B1 lamina was not dependent on carboxymethylation[190].

The B-type lamins are also involved in a wide range of nuclear functions, such as DNA replication and repair, and regulation of chromatin to regulate many cellular processes, including tissue development, cell cycle, cellular proliferation, senescence, and DNA damage response[171,191–192]. Hence, B-type lamins have also been linked to age-related organ dysfunctions and various human diseases, including disorders of the central nervous system and cancers[193].

## Type Ⅵ

The filamentous core of type Ⅵ IFs is composed of phakinin, whereas the beads contain filensin/phakinin hetero-oligomers[194]. AlphaA-crystallin forms a complex with filensin, phakinin, and a mixture of filensin and phakinin[195]. These IFs are localized at the periphery of the lens fiber cells and are believed to function in the maintenance of lens transparency[29,196]. Therefore, cataract occurs because of some mutations in filensin and phakinin[197].

*Filensin*

Filensin is a protein encoded by BFSP1[198]. Filensin binds specifically to lens vimentin. The tail B region (residues 370–416 of rat filensin, corresponding to 377–422 of human filensin) is essential for filament formation with phakinin. Therefore, the proteolytic cleavage-induced truncation of the tail B region within the cataract lens may potentially disrupt the formation of beaded filaments, consequently playing a role in the development of cataracts[199]. C-terminal BFSP1 fragments containing the myristoylation sequence colocalize with aquaporin 0 (AQP0) to regulate its water permeability[200].

*Phakinin*

Phakinin, also known as CP49, is a protein encoded by *BFSP2*[201]. Genetic deletion of BFSP2 in mice shows no obvious changes in lens architecture, with a subtle loss of optical clarity in the ocular lens, a loss that worsens with age[202].

## IF-associated proteins (IFAPs)

***Supplementary Table 2*** (available online) lists a number of proteins so far identified that may function

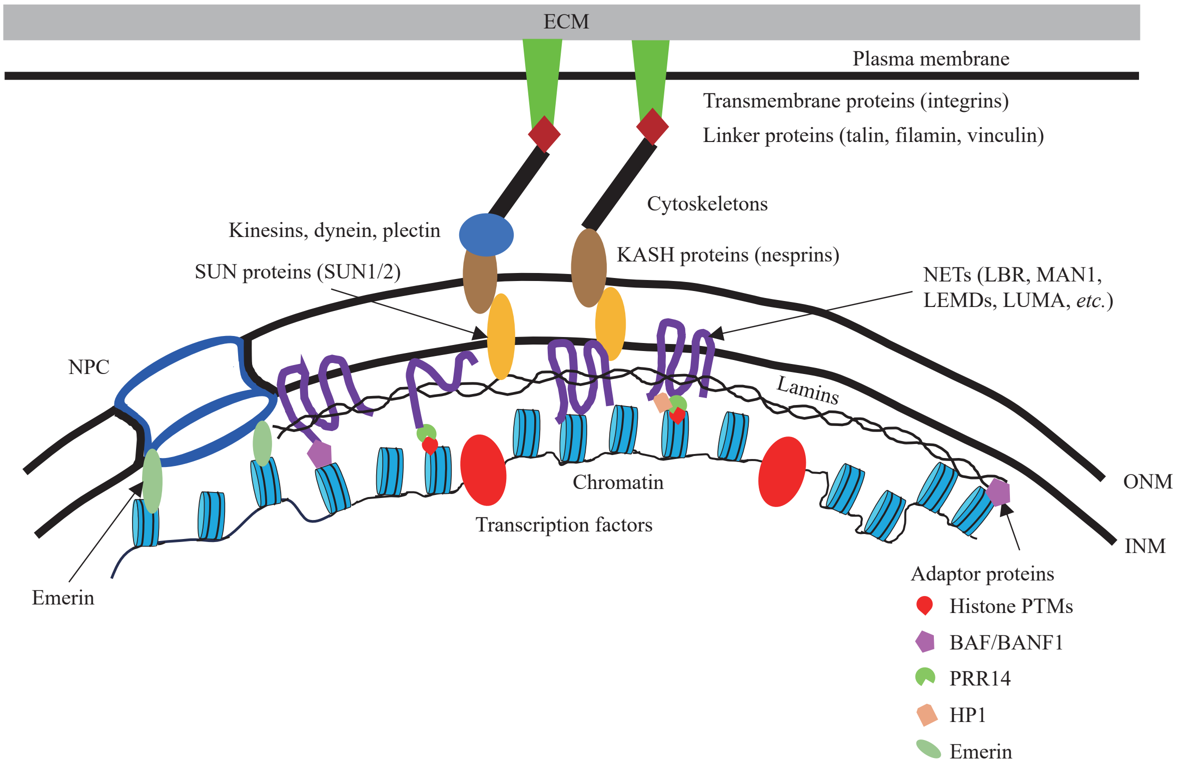


***Supplementary Fig. 4*** **The lamin-binding proteins-mediated connection between extracellular matrix (ECM) and chromatin.** The linker of nucleoskeleton and cytoskeleton complex is formed of KASH and SUN domain proteins. KASH domain proteins directly bind to cytoskeletons, whereas SUN domain proteins directly bind to nuclear lamins or indirectly through NE transmembrane proteins (NETs). The NETs directly bind to chromatin or indirectly through adaptor proteins, such as BAF/BANF1. Lamins also directly bind to chromatin or indirectly through the adaptor proteins. The nuclear pore complexes are also associated with chromatin through emerin. Emerin plays a key role in maintaining nuclear envelope integrity and regulating gene expression, and its mutations are associated with EDMD. Abbreviations: KASH, Klarsicht, ANC-1, Syne homology; SUN: Sad1 and UNC-84; LBR, lamin-B receptor, MAN1, an inner nuclear membrane protein; LEMDs, LEM domain-containing proteins; LUMA, transmembrane protein 43 (also known as TMEM43); BAF/BANF1, barrier-to-autointegration factor 1; PRR14, proline-rich protein 14; HP1, heterochromatin protein 1; LEM, LAP2-emerin-MAN1.

as IFAPs (307 in total and note that ***Supplementary Table 2*** includes components of the linkers of nucleoskeleton and cytoskeleton [LINC] complex, two of which have not been reported as IFAPs). In the "Structure" column, the domains of IFAPs that interact with IFs are described, and the Protein Data Bank accession numbers are indicated, while the "Binding IFs" column specifies the domains of IFs that bind to IFAPs, when known. Lagging behind the other cytoskeletal proteins[87–88], the discovery of IFAPs began in the 1980s, peaked around 2000, and has decreased since then, though research remains ongoing (***Supplementary Fig. 5***). In some cases, however, only simple biochemical properties and tissue localization using indirect immunofluorescence techniques or immunoprecipitation have been analyzed. These methods do not exclude the possibility of indirect interactions, and only 131 IFAPs have been definitively shown to directly interact with IFs. Additionally, little data is available on the molecular mechanisms by which they interact with IF. Nevertheless, this review assigned these IFAPs into different functional classes as follows.

**Plakin protein family**

The plakin protein family (7) shares a conserved plakin domain located near their N-terminus plakin domain and/or plakin repeat domains (PRDs) in their C-terminus[203–204]. Besides these plakin-related domains, some plakin family proteins consist of a tandem calponin-homology domain that is capable of binding to actin filaments, a coiled-coil domain, a linker subdomain, spectrin repeats, EF-hands, a Gas2-related homology domain, and a domain containing a series of glycine-serine-arginine repeats. Through these functional domains, plakin family proteins act as crosslinkers and scaffolds for other proteins[204]. For example, plectin cross-links IFs to MTs and actin filaments and attaches IFs to cell-cell and cell-matrix junctional complexes, such as hemidesmosomes, desmosomes, Z-lines, and focal contacts (***Supplementary Fig. 6***)[205]. Various isoforms of plakin proteins are expressed in different cell types with different short N-termini, enabling these isoforms

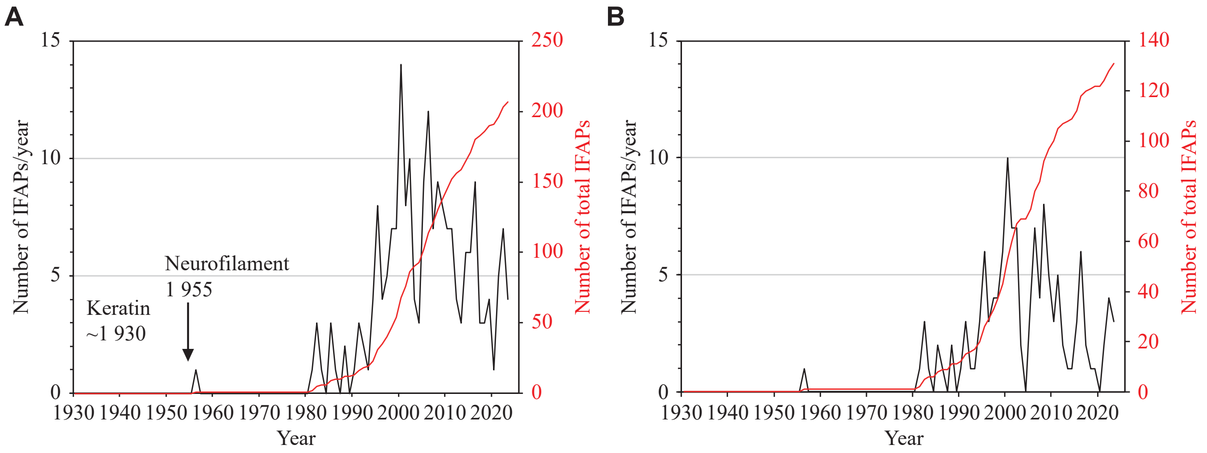


***Supplementary Fig. 5*** **The number of intermediate filament-associated proteins (IFAPs) published by year.** A: The number of IFAPs published by year (307 in total). Note that keratin-associated proteins (KRTAPs; 101 in total), except for KRTAP8-1, are not included in the graph because their genes were identified during the human genome project but have not been characterized. B: The number of IFAPs whose direct interactions with intermediate filaments were confirmed (131 in total).

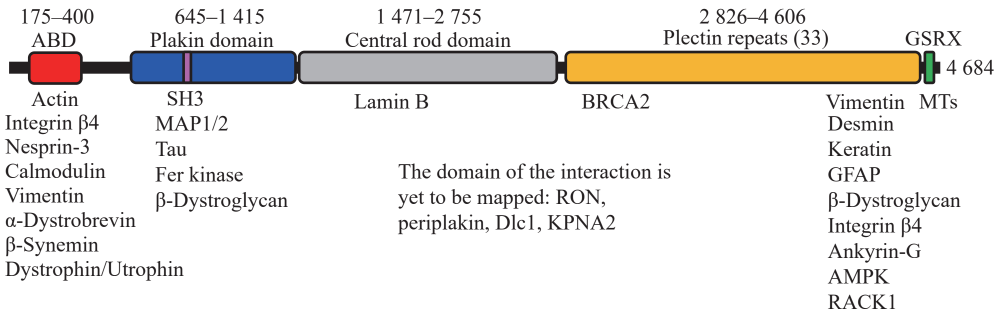


***Supplementary Fig. 6*** **Schematic structure of plectin and the binding regions for its binding partners.**

to specifically associate with distinct cellular structures[206–207]. For example, plectin 1a (P1a) isoform binds to integrin α6β4 in hemidesmosomes of keratinocytes, mediating stable anchorage of keratinocytes by linking the intracellular IF network system to ECM[208]. P1b isoform is mainly expressed in connective tissue as well as other types of cells and carries a mitochondrial targeting and anchoring signal in its N-terminal isoform-specific sequence[209]. Therefore, P1b inserts into the outer membrane of the mitochondria and connects it to the IF network. In primary keratinocytes, P1c is expressed as a second major plectin isoform and has a destabilizing effect on keratinocyte MTs[210].

Plectin interacts with various IF proteins, such as vimentin, neurofilaments, GFAP, and keratin[211]. The "linker" region between PRDs 5 and 6 in the C-terminal region of plectin is essential, but not sufficient, for the interaction with IFs[212]. The plectin-mediated bridge contributes to the keratinization of the mammalian skin[212]. In addition, this connection is necessary for the macroautophagic process under oxidative stress conditions[213]. Plectin-deficient fibroblasts exhibited abnormalities in their actin cytoskeleton and impaired migration[214–215].

In desmosomes, desmoplakin is essential for cell-cell adhesion. Humans have a single desmoplakin gene, but it is alternatively spliced to desmoplakin Ⅰ and desmoplakin Ⅱ. Desmoplakin Ⅰ associates with MT-binding proteins, while desmoplakin Ⅱ appears to be particularly important for epidermal adhesion[216–217].

Other plakin family proteins include dystonin (BPAG1), envoplakin, periplakin, epiplakin, and microtubule-actin cross-linking factor 1 (MACF1), but only BPAG1 and MACF1 contain actin-binding domains (***Supplementary Table 2***).

## The LINC complex and nuclear lamin-binding proteins (LBPs)

The LINC complex and nuclear LBPs (58 total and note that only 37 of them have been shown to directly bind to IFs) are important classes of proteins that regulate nuclear assembly, chromosome organization,

and even transcription[218–220]. Recent research has uncovered a wider and ever-growing range of functions, including roles in DNA repair, cell polarity, migration, and senescence[221–223]. The LINC complex consists of KASH domain proteins embedded in the outer nuclear membrane and SUN domain proteins in INM, with their interaction occurring within the NE lumen. Nesprins and lymphoid-restricted membrane protein (LRMP) are members of the KASH domain protein family (***Supplementary Fig. 4***). For example, nesprin-1 and nesprin-2 bind directly to actin filaments and indirectly to microtubules *via* motor proteins, whereas nesprin-3 binds to IFs through plectin, and nesprin-4 binds to MTs through kinesin[224–225]. KASH5 contains a KASH domain but lacks spectrin repeats, which excludes it from the nesprin family. It directly interacts with SUN1 and dynein, connecting telomeres to MTs to facilitate meiotic bouquet formation[226–228]. LRMP, also known as Jaw1, is primarily expressed in lymphoid tissues, involved in nuclear positioning and potentially regulating endoplasmic reticulum dynamics and lymphocyte function[229]. SUNs 1/2 directly bind to lamins, and SUN4 interacts with spermiogenesis-specific lamin B3[230–231]. However, direct interaction for SUN3 and SUN5 with lamins has not been demonstrated. Other NETs, such as LBR, emerin, and LEM domain-containing proteins, also interact with nuclear lamins. Furthermore, other LBPs, including novel NETs, are emerging from proteomic studies[232–233]. These studies revealed that some NETs are expressed ubiquitously, but many others show tissue-specific expression. Notably, only a small fraction of potential NETs have been experimentally shown to bind lamins directly[234].

Most LEM-domain proteins localize at INM, but some are also found in the endoplasmic reticulum or nuclear interior[235]. Some LEM-domain proteins have additional domains that bind DNA, lamins, or other chromatin proteins[236]. For example, lamin-associated polypeptide 2 beta (LAP2β) binds HA95, a chromatin protein involved in DNA replication[237]. All isoforms of LAP2 have a second "LEM-like" domain that directly binds DNA[238]. MAN1 also directly binds DNA through a carboxy-terminal winged helix domain[239]. Therefore, anchoring chromatin to the NE and lamins is highly complex but must be precisely organized and controlled, as this network regulates gene expression[240–241].

## Keratin-associated proteins (KAPs)

KAPs (101) are classified into three groups based on their characteristic amino acid sequences: high sulfur (HS) KAPs, ultra-high sulfur (UHS) KAPs, and high glycine-tyrosine (HGT) KAPs. There are 14 HS KAPs, with less than 30 mol.% cysteine; four UHS KAPs, with more than 30 mol.% cysteine; and seven HGT KAPs, with 35–60 mol.% glycine or tyrosine (***Supplementary Table 2***)[242]. However, some researchers do not include KRTAP28-1–8 as KAPs because they are regarded as pseudogenes[243]. While HS and UHS KAPs are known to function in disulfide bond crosslinking with keratins, the function of HGT KAPs is not well known. Nevertheless, KAP8.1, one of the HGT KAPs, interacts specifically with the head domain of KRT85[244]. A mixture of hair KRT35 and KRT85 IF fibers forms dispersed IFs, but the addition of KAP8.1 bundles the IFs, contributing to the mechanical robustness of hair. In addition, certain KAPs have a function in driving cell proliferation. For example, KAP2.3, an HS KAP, regulates transforming growth factor beta signaling to control cancer cell migration, metastasis, and proliferation[245]. However, the majority of KAPs have not been experimentally studied, and their functions remain unknown. Nevertheless, the abundance of genes related to keratins and KAPs suggests a significant contribution of keratin IFs to the evolution of vertebrates, particularly in terms of development and environmental adaptation.

## Other IFAPs

Other IFAPs (142) include a wide variety of proteins, such as chaperone, kinase, phosphatase, scaffold, crosslinker, integrin, and calcium-binding protein (***Supplementary Table 2***). For instance, vimentin filaments directly interact with and increase β3 integrin avidity by clustering integrins, thereby enhancing cell adhesion[246]. KRT17 interacts with TNF receptor 1 (TNFR1)-associated death domain protein, an essential adaptor for TNFR1 signaling, and they jointly regulate hair cycling[247]. Additionally, KRT6 interacts with Src *via* its SH2 domain, negatively regulating Src kinase activity and reducing the migratory potential of skin keratinocytes during wound healing[248]. However, direct interactions between some IFAPs listed in ***Supplementary Table 2*** and IFs have yet to be demonstrated, making it challenging to investigate the biological functions of these interactions[246].

We previously performed computational screening to identify new MT-associated proteins (MAPs) using the Human Protein Atlas database and a list of known MAPs similar to the one in ***Supplementary Table 2***[249]. The screening is based on the subcellular protein distribution data from the database. There are

numerous proteins (approximately 70) that colocalize with IFs but are not listed in ***Supplementary Table 2***, suggesting that these proteins are potential new IFAPs. We have recently conducted biochemical tests on some of these proteins and found that exogenously expressed BCAS4, TMEM81, EML4, and LHFPL2 colocalize with vimentin filaments in tissue culture cells but do not directly bind to purified vimentin (unpublished data). Therefore, many of the IFAPs listed in ***Supplementary Table 2*** require further validation.

## References


[1] Peter A, Stick R. Evolutionary aspects in intermediate filament proteins[J]. *Curr Opin Cell Biol*, 2015, 32: 48–55.

[2] Etienne-Manneville S. Cytoplasmic intermediate filaments in cell biology[J]. *Annu Rev Cell Dev Biol*, 2018, 34: 1–28.

[3] Dutour-Provenzano G, Etienne-Manneville S. Intermediate filaments[J]. *Curr Biol*, 2021, 31(10): R522–R529.

[4] Eldirany SA, Lomakin IB, Ho M, et al. Recent insight into intermediate filament structure[J]. *Curr Opin Cell Biol*, 2021, 68: 132–143.

[5] Sinha S. Regulation of intermediate filament gene expression[J]. *Methods Cell Biol*, 2004, 78: 267–296.

[6] Szeverenyi I, Cassidy AJ, Chung CW, et al. The human intermediate filament database: comprehensive information on a gene family involved in many human diseases[J]. *Hum Mutat*, 2008, 29(3): 351–360.

[7] Fraser RDB, Parry DAD. Structural hierarchy of trichocyte keratin intermediate filaments[M]//Plowman JE, Harland DP, Deb-Choudhury S. The Hair Fibre: Proteins, Structure and Development. Singapore: Springer, 2018: 57–70.

[8] Traweek ST, Liu J, Battifora H. Keratin gene expression in non-epithelial tissues. Detection with polymerase chain reaction[J]. *Am J Pathol*, 1993, 142(4): 1111–1118.

[9] Romano R, Del Fiore VS, Bucci C. Role of the intermediate filament protein peripherin in health and disease[J]. *Int J Mol Sci*, 2022, 23(23): 15416.

[10] Pajares MA, Pérez-Sala D. Type Ⅲ intermediate filaments in redox interplay: key role of the conserved cysteine residue[J]. *Biochem Soc Trans*, 2024, 52(2): 849–860.

[11] Kotaich F, Caillol D, Bomont P. Neurofilaments in health and Charcot-Marie-Tooth disease[J]. *Front Cell Dev Biol*, 2023, 11: 1275155.

[12] van Asperen JV, Kotaich F, Caillol D, et al. Neurofilaments: novel findings and future challenges[J]. *Curr Opin Cell Biol*, 2024, 87: 102326.

[13] Hol EM, Capetanaki Y. Type Ⅲ intermediate filaments desmin, glial fibrillary acidic protein (GFAP), vimentin, and peripherin[J]. *Cold Spring Harb Perspect Biol*, 2017, 9(12): a021642.

[14] Lazarides E, Baizer DR Jr. Specificity of desmin to avian and mammalian muscle cells[J]. *Cell*, 1978, 14(2): 429–438.

[15] Newey SE, Howman EV, Ponting CP, et al. Syncoilin, a novel member of the intermediate filament superfamily that interacts with α-dystrobrevin in skeletal muscle[J]. *J Biol Chem*, 2001, 276(9): 6645–6655.

[16] Jessen KR, Mirsky R. Glial cells in the enteric nervous system contain glial fibrillary acidic protein[J]. *Nature*, 1980, 286(5774): 736–377.

[17] Ding EA, Kumar S. Neurofilament biophysics: from structure to biomechanics[J]. *Mol Biol Cell*, 2024, 35(5): re1.

[18] Yuan A, Rao MV, Sasaki T, et al. α-internexin is structurally and functionally associated with the neurofilament triplet proteins in the mature CNS[J]. *J Neurosci*, 2006, 26(39): 10006–10019.

[19] Michalczyk K, Ziman M. Nestin structure and predicted function in cellular cytoskeletal organisation[J]. *Histol Histopathol*, 2005, 20(2): 665–671.

[20] Paulin D, Hovhannisyan Y, Kasakyan S, et al. Synemin-related skeletal and cardiac myopathies: an overview of pathogenic variants[J]. *Am J Physiol Cell Physiol*, 2020, 318(4): C709–C718.

[21] Paul M, Skalli O. Synemin: molecular features and the use of proximity ligation assay to study its interactions[J]. *Methods Enzymol*, 2016, 568: 537–555.

[22] Aebi U, Cohn J, Buhle L, et al. The nuclear lamina is a meshwork of intermediate-type filaments[J]. *Nature*, 1986, 323(6088): 560–564.

[23] Barboro P, D'Arrigo C, Repaci E, et al. Organization of the lamin scaffold in the internal nuclear matrix of normal and transformed hepatocytes[J]. *Exp Cell Res*, 2010, 316(6): 992–1001.

[24] Sapra KT, Qin Z, Dubrovsky-Gaupp A, et al. Nonlinear mechanics of lamin filaments and the meshwork topology build an emergent nuclear lamina[J]. *Nat Commun*, 2020, 11(1): 6205.

[25] Sapra KT, Medalia O. Bend, push, stretch: remarkable structure and mechanics of single intermediate filaments and meshworks[J]. *Cells*, 2021, 10(8): 1960.

[26] Peter A, Stick R. Evolution of the lamin protein family: what introns can tell[J]. *Nucleus*, 2012, 3(1): 44–59.

[27] Zheng M, Jin G, Zhou Z. Post-translational modification of lamins: mechanisms and functions[J]. *Front Cell Dev Biol*, 2022, 10: 864191.

[28] Sobo JM, Alagna NS, Sun SX, et al. Lamins: the backbone of the nucleocytoskeleton interface[J]. *Curr Opin Cell Biol*, 2024, 86: 102313.

[29] Song S, Landsbury A, Dahm R, et al. Functions of the intermediate filament cytoskeleton in the eye lens[J]. *J Clin Invest*, 2009, 119(7): 1837–1848.

[30] Herrmann H, Aebi U. Intermediate filaments: molecular structure, assembly mechanism, and integration into functionally distinct intracellular scaffolds[J]. *Annu Rev Biochem*, 2004, 73: 749–789.

[31] Ahn J, Jo I, Kang SM, et al. Structural basis for lamin

assembly at the molecular level[J]. *Nat Commun*, 2019, 10(1): 3757.

[32] Lee CH, Kim MS, Li S, et al. Structure-function analyses of a keratin heterotypic complex identify specific keratin regions involved in intermediate filament assembly[J]. *Structure*, 2020, 28(3): 355–362.e4.

[33] Hanukoglu I, Fuchs E. The cDNA sequence of a Type Ⅱ cytoskeletal keratin reveals constant and variable structural domains among keratins[J]. *Cell*, 1983, 33(3): 915–924.

[34] Steinert PM, Jones JCR, Goldman RD. Intermediate filaments[J]. *J Cell Biol*, 1984, 99(1 Pt 2): 22s–27s.

[35] Parry DAD. Hendecad repeat in segment 2A and linker L2 of intermediate filament chains implies the possibility of a right-handed coiled-coil structure[J]. *J Struct Biol*, 2006, 155(2): 370–374.

[36] Nicolet S, Herrmann H, Aebi U, et al. Atomic structure of vimentin coil 2[J]. *J Struct Biol*, 2010, 170(2): 369–376.

[37] Chamcheu JC, Siddiqui IA, Syed DN, et al. Keratin gene mutations in disorders of human skin and its appendages[J]. *Arch Biochem Biophys*, 2011, 508(2): 123–137.

[38] Traub P, Kühn S, Grüb S. Separation and characterization of homo and hetero-oligomers of the intermediate filament proteins desmin and vimentin[J]. *J Mol Biol*, 1993, 230(3): 837–856.

[39] Sokolova AV, Kreplak L, Wedig T, et al. Monitoring intermediate filament assembly by small-angle x-ray scattering reveals the molecular architecture of assembly intermediates[J]. *Proc Natl Acad Sci U S A*, 2006, 103(44): 16206–16211.

[40] Kang DS, Moriarty A, Wang YJ, et al. Ectopic expression of a truncated isoform of hair keratin 81 in breast cancer alters biophysical characteristics to promote metastatic propensity[J]. *Adv Sci (Weinh)*, 2024, 11(5): 2300509.

[41] Kirmse R, Bouchet-Marquis C, Page C, et al. Three-dimensional cryo-electron microscopy on intermediate filaments[J]. *Methods Cell Biol*, 2010, 96: 565–589.

[42] Steinert PM, Marekov LN, Parry DA. Diversity of intermediate filament structure. Evidence that the alignment of coiled-coil molecules in vimentin is different from that in keratin intermediate filaments[J]. *J Biol Chem*, 1993, 268(33): 24916–24925.

[43] Steinert PM, Marekov LN, Parry DAD. Conservation of the structure of keratin intermediate filaments: molecular mechanism by which different keratin molecules integrate into preexisting keratin intermediate filaments during differentiation[J]. *Biochemistry*, 1993, 32(38): 10046–10056.

[44] Lilina AV, Chernyatina AA, Guzenko D, et al. Lateral $A_{11}$ type tetramerization in lamins[J]. *J Struct Biol*, 2020, 209(1): 107404.

[45] Hughes MP, Sawaya MR, Boyer DR, et al. Atomic structures of low-complexity protein segments reveal kinked β sheets that assemble networks[J]. *Science*, 2018, 359(6376): 698–701.

[46] Dey R, Burkhard P. A proposed atomic model of the head-to-tail interaction in the filament structure of vimentin[J]. *J Biomol Struct Dyn*, 2020, 38(16): 4921–4927.

[47] Stalmans G, Lilina AV, Vermeire PJ, et al. Addressing the molecular mechanism of longitudinal lamin assembly using chimeric fusions[J]. *Cells*, 2020, 9(7): 1633.

[48] Zhou X, Kato M, McKnight SL. How do disordered head domains assist in the assembly of intermediate filaments?[J]. *Curr Opin Cell Biol*, 2023, 85: 102262.

[49] Lee CH, Kim MS, Chung BM, et al. Structural basis for heteromeric assembly and perinuclear organization of keratin filaments[J]. *Nat Struct Mol Biol*, 2012, 19(7): 707–715.

[50] Eldirany SA, Ho M, Hinbest AJ, et al. Human keratin 1/10–1B tetramer structures reveal a knob-pocket mechanism in intermediate filament assembly[J]. *EMBO J*, 2019, 38(11): e100741.

[51] Eibauer M, Weber MS, Kronenberg-Tenga R, et al. Vimentin filaments integrate low-complexity domains in a complex helical structure[J]. *Nat Struct Mol Biol*, 2024, 31(6): 939–949.

[52] Ho M, Thompson B, Fisk JN, et al. Update of the keratin gene family: evolution, tissue-specific expression patterns, and relevance to clinical disorders[J]. *Hum Genomics*, 2022, 16(1): 1.

[53] Kalabusheva EP, Shtompel AS, Rippa AL, et al. A kaleidoscope of keratin gene expression and the mosaic of its regulatory mechanisms[J]. *Int J Mol Sci*, 2023, 24(6): 5603.

[54] Di Russo J, Magin TM, Leube RE. A keratin code defines the textile nature of epithelial tissue architecture[J]. *Curr Opin Cell Biol*, 2023, 85: 102236.

[55] Deo PN, Deshmukh R. Pathophysiology of keratinization[J]. *J Oral Maxillofac Pathol*, 2018, 22(1): 86–91.

[56] Rogel MR, Jaitovich A, Ridge KM. The role of the ubiquitin proteasome pathway in keratin intermediate filament protein degradation[J]. *Proc Am Thorac Soc*, 2010, 7(1): 71–76.

[57] Jacob JT, Coulombe PA, Kwan R, et al. Types Ⅰ and Ⅱ keratin intermediate filaments[J]. *Cold Spring Harb Perspect Biol*, 2018, 10(4): a018275.

[58] Ku NO, Omary MB. Phosphorylation of human keratin 8 *in vivo* at conserved head domain serine 23 and at epidermal growth factor-stimulated tail domain serine 431[J]. *J Biol Chem*, 1997, 272(11): 7556–7564.

[59] Liao J, Ku NO, Omary MB. Stress, apoptosis, and mitosis induce phosphorylation of human keratin 8 at Ser-73 in tissues and cultured cells[J]. *J Biol Chem*, 1997, 272(28): 17565–17573.

[60] Chung BM, Rotty JD, Coulombe PA. Networking galore: intermediate filaments and cell migration[J]. *Curr Opin Cell Biol*, 2013, 25(5): 600–612.

[61] Miao Q, Xu Y, Yin H, et al. KRT8 phosphorylation regulates the epithelial-mesenchymal transition in retinal pigment epithelial cells through autophagy modulation[J]. *J Cell Mol Med*, 2020, 24(5): 3217–3228.

[62] Wang D, Shang Q, Mao J, et al. Phosphorylation of KRT8

(keratin 8) by excessive mechanical load-activated PKN (protein kinase N) impairs autophagosome initiation and contributes to disc degeneration[J]. *Autophagy*, 2023, 19(9): 2485–2503.

[63] Zhou Q, Snider NT, Liao J, et al. Characterization of *in vivo* keratin 19 phosphorylation on tyrosine-391[J]. *PLoS One*, 2010, 5(10): e13538.

[64] Velez-delValle C, Marsch-Moreno M, Castro-Muñozledo F, et al. Epithelial cell migration requires the interaction between the vimentin and keratin intermediate filaments[J]. *Sci Rep*, 2016, 6: 24389.

[65] Pondel MD, King ML. Localized maternal mRNA related to transforming growth factor $\beta$ mRNA is concentrated in a cytokeratin-enriched fraction from *Xenopus* oocytes[J]. *Proc Natl Acad Sci U S A*, 1988, 85(20): 7612–7616.

[66] Challa AA, Stefanovic B. A novel role of vimentin filaments: binding and stabilization of collagen mRNAs[J]. *Mol Cell Biol*, 2011, 31(18): 3773–3789.

[67] Schmidt Y, Biniossek M, Stark GB, et al. Osteoblastic alkaline phosphatase mRNA is stabilized by binding to vimentin intermediary filaments[J]. *Biol Chem*, 2015, 396(3): 253–260.

[68] Fallatah A, Anastasakis DG, Manzourolajdad A, et al. Keratin 19 binds and regulates cytoplasmic HNRNPK mRNA targets in triple-negative breast cancer[J]. *BMC Mol Cell Biol*, 2023, 24(1): 26.

[69] Coulombe PA, Pineda CM, Jacob JT, et al. Nuclear roles for non-lamin intermediate filament proteins[J]. *Curr Opin Cell Biol*, 2024, 86: 102303.

[70] Franke WW, Schmid E, Osborn M, et al. Different intermediate-sized filaments distinguished by immunofluorescence microscopy[J]. *Proc Natl Acad Sci U S A*, 1978, 75(10): 5034–5038.

[71] Paulin D, Lilienbaum A, Kardjian S, et al. Vimentin: regulation and pathogenesis[J]. *Biochimie*, 2022, 197: 96–112.

[72] Snider NT, Omary MB. Assays for posttranslational modifications of intermediate filament proteins[J]. *Methods Enzymol*, 2016, 568: 113–138.

[73] Eriksson JE, He T, Trejo-Skalli AV, et al. Specific *in vivo* phosphorylation sites determine the assembly dynamics of vimentin intermediate filaments[J]. *J Cell Sci*, 2004, 117(Pt 6): 919–932.

[74] Sihag RK, Inagaki M, Yamaguchi T, et al. Role of phosphorylation on the structural dynamics and function of types Ⅲ and Ⅳ intermediate filaments[J]. *Exp Cell Res*, 2007, 313(10): 2098–2109.

[75] Llorente-González C, González-Rodriguez M, Vicente-Manzanares M. Targeting cytoskeletal phosphorylation in cancer[J]. *Explor Target Antitumor Ther*, 2021, 2(3): 292–308.

[76] Goel RK, Paczkowska M, Reimand J, et al. Phosphoproteomics analysis identifies novel candidate substrates of the nonreceptor tyrosine kinase, *S*rc-related kinase lacking c-terminal regulatory tyrosine and N-terminal *M*yristoylation sites (SRMS)[J]. *Mol Cell Proteomics*, 2018, 17(5): 925–947.

[77] Ratnayake WS, Apostolatos CA, Breedy S, et al. Atypical PKCs activate vimentin to facilitate prostate cancer cell motility and invasion[J]. *Cell Adh Migr*, 2021, 15(1): 37–57.

[78] Ikawa K, Satou A, Fukuhara M, et al. Inhibition of endocytic vesicle fusion by Plk1-mediated phosphorylation of vimentin during mitosis[J]. *Cell Cycle*, 2014, 13(1): 126–137.

[79] MacTaggart B, Kashina A. Posttranslational modifications of the cytoskeleton[J]. *Cytoskeleton (Hoboken)*, 2021, 78(4): 142–173.

[80] Guo D, Song X, Guo T, et al. Vimentin acetylation is involved in SIRT5-mediated hepatocellular carcinoma migration[J]. *Am J Cancer Res*, 2018, 8(12): 2453–2466.

[81] Renganathan B, Zewe JP, Cheng Y, et al. Gigaxonin is required for intermediate filament transport[J]. *FASEB J*, 2023, 37(5): e22886.

[82] Mahammad S, Murthy SNP, Didonna A, et al. Giant axonal neuropathy-associated gigaxonin mutations impair intermediate filament protein degradation[J]. *J Clin Invest*, 2013, 123(5): 1964–1975.

[83] Brentville VA, Metheringham RL, Gunn B, et al. Citrullinated vimentin presented on MHC-Ⅱ in tumor cells is a target for $CD4^{+}$ T-cell-mediated antitumor immunity[J]. *Cancer Res*, 2016, 76(3): 548–560.

[84] Ishigami A, Ohsawa T, Hiratsuka M, et al. Abnormal accumulation of citrullinated proteins catalyzed by peptidylarginine deiminase in hippocampal extracts from patients with Alzheimer's disease[J]. *J Neurosci Res*, 2005, 80(1): 120–128.

[85] Wizeman JW, Nicholas AP, Ishigami A, et al. Citrullination of glial intermediate filaments is an early response in retinal injury[J]. *Mol Vis*, 2016, 22: 1137–1155.

[86] Chang L, Goldman RD. Intermediate filaments mediate cytoskeletal crosstalk[J]. *Nat Rev Mol Cell Biol*, 2004, 5(8): 601–613.

[87] Gao J, Nakamura F. Actin-associated proteins and small molecules targeting the actin cytoskeleton[J]. *Int J Mol Sci*, 2022, 23(4): 2118.

[88] Peng N, Nakamura F. Microtubule-associated proteins and enzymes modifying tubulin[J]. *Cytoskeleton (Hoboken)*, 2023, 80(3-4): 60–76.

[89] Schaedel L, Lorenz C, Schepers AV, et al. Vimentin intermediate filaments stabilize dynamic microtubules by direct interactions[J]. *Nat Commun*, 2021, 12(1): 3799.

[90] Wu H, Shen Y, Sivagurunathan S, et al. Vimentin intermediate filaments and filamentous actin form unexpected interpenetrating networks that redefine the cell cortex[J]. *Proc Natl Acad Sci U S A*, 2022, 119(10): e2115217119.

[91] Ridge KM, Eriksson JE, Pekny M, et al. Roles of vimentin in health and disease[J]. *Genes Dev*, 2022, 36(7-8): 391–407.

[92] Bucki R, Iwamoto DV, Shi X, et al. Extracellular vimentin

is sufficient to promote cell attachment, spreading, and motility by a mechanism involving N-acetyl glucosamine-containing structures[J]. *J Biol Chem*, 2023, 299(8): 104963.
[93] Parvanian S, Coelho-Rato LS, Eriksson JE, et al. The molecular biophysics of extracellular vimentin and its role in pathogen-host interactions[J]. *Curr Opin Cell Biol*, 2023, 85: 102233.
[94] Thalla DG, Lautenschläger F. Extracellular vimentin: Battle between the devil and the angel[J]. *Curr Opin Cell Biol*, 2023, 85: 102265.
[95] Ceschi S, Berselli M, Cozzaglio M, et al. Vimentin binds to G-quadruplex repeats found at telomeres and gene promoters[J]. *Nucleic Acids Res*, 2022, 50(3): 1370–1381.
[96] Lazarides E, Hubbard BD. Immunological characterization of the subunit of the 100 A filaments from muscle cells[J]. *Proc Natl Acad Sci U S A*, 1976, 73(12): 4344–4348.
[97] Agnetti G, Herrmann H, Cohen S. New roles for desmin in the maintenance of muscle homeostasis[J]. *FEBS J*, 2022, 289(10): 2755–2770.
[98] Su W, van Wijk SW, Brundel BJJM. Desmin variants: trigger for cardiac arrhythmias?[J]. *Front Cell Dev Biol*, 2022, 10: 986718.
[99] Agbulut O, Li Z, Mouly V, et al. Analysis of skeletal and cardiac muscle from desmin knock-out and normal mice by high resolution separation of myosin heavy-chain isoforms[J]. *Biol Cell*, 1996, 88(3): 131–135.
[100] Li Z, Colucci-Guyon E, Pinçon-Raymond M, et al. Cardiovascular lesions and skeletal myopathy in mice lacking desmin[J]. *Dev Biol*, 1996, 175(2): 362–366.
[101] Papadopoulos C, Malfatti E, Métay C, et al. Deep characterization of a Greek patient with desmin-related myofibrillar myopathy and cardiomyopathy[J]. *Int J Mol Sci*, 2023, 24(13): 11181.
[102] West G, Sedighi S, Agnetti G, et al. Intermediate filaments in the heart: the dynamic duo of desmin and lamins orchestrates mechanical force transmission[J]. *Curr Opin Cell Biol*, 2023, 85: 102280.
[103] Aweida D, Rudesky I, Volodin A, et al. GSK3-β promotes calpain-1-mediated desmin filament depolymerization and myofibril loss in atrophy[J]. *J Cell Biol*, 2018, 217(10): 3698–3714.
[104] Bouvet M, Dubois-Deruy E, Turkieh A, et al. Desmin aggrephagy in rat and human ischemic heart failure through PKCζ and GSK3β as upstream signaling pathways[J]. *Cell Death Discov*, 2021, 7(1): 153.
[105] Winter L, Unger A, Berwanger C, et al. Imbalances in protein homeostasis caused by mutant desmin[J]. *Neuropathol Appl Neurobiol*, 2019, 45(5): 476–494.
[106] Kedia N, Arhzaouy K, Pittman SK, et al. Desmin forms toxic, seeding-competent amyloid aggregates that persist in muscle fibers[J]. *Proc Natl Acad Sci U S A*, 2019, 116(34): 16835–16840.
[107] Ishizaka T, Hatori K. Direct observation of oriented behavior of actin filaments interacting with desmin intermediate filaments[J]. *Biochim Biophys Acta Gen Subj*, 2023, 1867(12): 130488.
[108] Kemp MW, Edwards B, Burgess M, et al. Syncoilin isoform organization and differential expression in murine striated muscle[J]. *J Struct Biol*, 2009, 165(3): 196–203.
[109] Moorwood C. Syncoilin, an intermediate filament-like protein linked to the dystrophin associated protein complex in skeletal muscle[J]. *Cell Mol Life Sci*, 2008, 65(19): 2957–2963.
[110] Clarke WT, Edwards B, McCullagh KJA, et al. Syncoilin modulates peripherin filament networks and is necessary for large-calibre motor neurons[J]. *J Cell Sci*, 2010, 123(Pt 15): 2543–2552.
[111] Zhang J, Bang ML, Gokhin DS, et al. Syncoilin is required for generating maximum isometric stress in skeletal muscle but dispensable for muscle cytoarchitecture[J]. *Am J Physiol Cell Physiol*, 2008, 294(5): C1175–C1182.
[112] Portier MM, de Néchaud B, Gros F. Peripherin, a new member of the intermediate filament protein family[J]. *Dev Neurosci*, 1983, 6(6): 335–344.
[113] Larivière RC, Nguyen MD, Ribeiro-da-Silva A, et al. Reduced number of unmyelinated sensory axons in peripherin null mice[J]. *J Neurochem*, 2002, 81(3): 525–532.
[114] Cederholm JME, Parley KE, Perera CJ, et al. Noise-induced hearing loss vulnerability in type Ⅲ intermediate filament peripherin gene knockout mice[J]. *Front Neurol*, 2022, 13: 962227.
[115] Cogli L, Progida C, Thomas CL, et al. Charcot-Marie-Tooth type 2B disease-causing RAB7A mutant proteins show altered interaction with the neuronal intermediate filament peripherin[J]. *Acta Neuropathol*, 2013, 125(2): 257–272.
[116] Eng LF, Vanderhaeghen JJ, Bignami A, et al. An acidic protein isolated from fibrous astrocytes[J]. *Brain Res*, 1971, 28(2): 351–354.
[117] Uyeda CT, Eng LF, Bignami A. Immunological study of the glial fibrillary acidic protein[J]. *Brain Res*, 1972, 37(1): 81–89.
[118] van Asperen JV, Robe P, Hol EM. GFAP alternative splicing and the relevance for disease - a focus on diffuse gliomas[J]. *ASN Neuro*, 2022, 14: 17590914221102065.
[119] Phillips CL, Faridounnia M, Armao D, et al. Stability dynamics of neurofilament and GFAP networks and protein fragments[J]. *Curr Opin Cell Biol*, 2023, 85: 102266.
[120] Inagaki M, Nakamura Y, Takeda M, et al. Glial fibrillary acidic protein: dynamic property and regulation by phosphorylation[J]. *Brain Pathol*, 1994, 4(3): 239–243.
[121] Moeton M, Stassen OMJA, Sluijs JA, et al. GFAP isoforms control intermediate filament network dynamics, cell morphology, and focal adhesions[J]. *Cell Mol Life Sci*, 2016, 73(21): 4101–4120.
[122] van Asperen JV, Fedorushkova DM, Robe PAJT, et al. Investigation of glial fibrillary acidic protein (GFAP) in body fluids as a potential biomarker for glioma: a systematic review and meta-analysis[J]. *Biomarkers*, 2022, 27(1): 1–12.
[123] Lin J, Dong L, Yu L, et al. Autoimmune glial fibrillary

acidic protein astrocytopathy coexistent with reversible splenial lesion syndrome: a case report and literature review[J]. *Front Neurol*, 2023, 14: 1192118.
[124] Kim H, Lee EJ, Lim YM, et al. Glial fibrillary acidic protein in blood as a disease biomarker of neuromyelitis optica spectrum disorders[J]. *Front Neurol*, 2022, 13: 865730.
[125] Tan C, Zhong M, Yao Z, et al. Anti-GFAP antibody-associated hypertrophic pachymeningitis[J]. *Neuropediatrics*, 2022, 53(2): 143–145.
[126] Zottel A, Jovčevska I, Šamec N, et al. Cytoskeletal proteins as glioblastoma biomarkers and targets for therapy: a systematic review[J]. *Crit Rev Oncol Hematol*, 2021, 160: 103283.
[127] Heins S, Wong PC, Müller S, et al. The rod domain of NF-L determines neurofilament architecture, whereas the end domains specify filament assembly and network formation[J]. *J Cell Biol*, 1993, 123(6 Pt 1): 1517–1533.
[128] Lee MK, Xu Z, Wong PC, et al. Neurofilaments are obligate heteropolymers *in vivo*[J]. *J Cell Biol*, 1993, 122(6): 1337–1350.
[129] Yuan A, Rao MV, Veeranna, et al. Neurofilaments and neurofilament proteins in health and disease[J]. *Cold Spring Harb Perspect Biol*, 2017, 9(4): a018309.
[130] Dong DL, Xu Z, Chevrier MR, et al. Glycosylation of mammalian neurofilaments. Localization of multiple O-linked N-acetylglucosamine moieties on neurofilament polypeptides L and M[J]. *J Biol Chem*, 1993, 268(22): 16679–16687.
[131] Deng Y, Li B, Liu F, et al. Regulation between O-GlcNAcylation and phosphorylation of neurofilament-M and their dysregulation in Alzheimer disease[J]. *FASEB J*, 2008, 22(1): 138–145.
[132] Lüdemann N, Clement A, Hans VH, et al. O-glycosylation of the tail domain of neurofilament protein M in human neurons and in spinal cord tissue of a rat model of amyotrophic lateral sclerosis (ALS)[J]. *J Biol Chem*, 2005, 280(36): 31648–31658.
[133] Pant HC, Veeranna, Grant P. Regulation of axonal neurofilament phosphorylation[J]. *Curr Top Cell Regul*, 2001, 36: 133–150.
[134] Maccioni RB, Otth C, Concha II, et al. The protein kinase Cdk5. Structural aspects, roles in neurogenesis and involvement in Alzheimer's pathology[J]. *Eur J Biochem*, 2001, 268(6): 1518–1527.
[135] Gordon BA. Neurofilaments in disease: what do we know?[J]. *Curr Opin Neurobiol*, 2020, 61: 105–115.
[136] Doganyigit Z, Eroglu E, Okan A. Intermediate filament proteins are reliable immunohistological biomarkers to help diagnose multiple tissue-specific diseases[J]. *Anat Histol Embryol*, 2023, 52(5): 655–672.
[137] Pachter JS, Liem RK. alpha-internexin, a 66-kD intermediate filament-binding protein from mammalian central nervous tissues[J]. *J Cell Biol*, 1985, 101(4): 1316–1322.
[138] Zhao J, Liem RKH. α-internexin and peripherin: expression, assembly, functions, and roles in disease[J]. *Methods Enzymol*, 2016, 568: 477–507.
[139] Wang Y, Chen Y, Li X, et al. Loss of expression and prognosis value of alpha-internexin in gastroentero-pancreatic neuroendocrine neoplasm[J]. *BMC Cancer*, 2018, 18(1): 691.
[140] Bott CJ, Winckler B. Intermediate filaments in developing neurons: beyond structure[J]. *Cytoskeleton (Hoboken)*, 2020, 77(3-4): 110–128.
[141] Willoughby V, Sonawala A, Werlang-Perurena A, et al. A comparative immunohistochemical analysis of small round cell tumors of childhood: utility of peripherin and α-internexin as markers for neuroblastomas[J]. *Appl Immunohistochem Mol Morphol*, 2008, 16(4): 344–348.
[142] Yuan A, Nixon RA. Neurofilament proteins as biomarkers to monitor neurological diseases and the efficacy of therapies[J]. *Front Neurosci*, 2021, 15: 689938.
[143] Sabbatini D, Raggi F, Ruggero S, et al. Evaluation of peripherin in biofluids of patients with motor neuron diseases[J]. *Ann Clin Transl Neurol*, 2021, 8(8): 1750–1754.
[144] Abumuhor IA, Spencer PH, Cohlberg JA. The pathway of assembly of intermediate filaments from recombinant α-internexin[J]. *J Struct Biol*, 1998, 123(3): 187–198.
[145] Tanaka J, Ogawara M, Ando S, et al. Phosphorylation of a 62 kd porcine α-internexin, a newly identified intermediate filament protein[J]. *Biochem Biophys Res Commun*, 1993, 196(1): 115–123.
[146] Li Y, Bai L, Yu H, et al. Epigenetic inactivation of α-internexin accelerates microtubule polymerization in colorectal cancer[J]. *Cancer Res*, 2020, 80(23): 5203–5215.
[147] Lendahl U, Zimmerman LB, McKay RDG. CNS stem cells express a new class of intermediate filament protein[J]. *Cell*, 1990, 60(4): 585–595.
[148] Bernal A, Arranz L. Nestin-expressing progenitor cells: function, identity and therapeutic implications[J]. *Cell Mol Life Sci*, 2018, 75(12): 2177–2195.
[149] Yamagishi A, Susaki M, Takano Y, et al. The structural function of nestin in cell body softening is correlated with cancer cell metastasis[J]. *Int J Biol Sci*, 2019, 15(7): 1546–1556.
[150] Esue O, Carson AA, Tseng Y, et al. A direct interaction between actin and vimentin filaments mediated by the tail domain of vimentin[J]. *J Biol Chem*, 2006, 281(41): 30393–30399.
[151] Granger BL, Lazarides E. Synemin: a new high molecular weight protein associated with desmin and vimentin filaments in muscle[J]. *Cell*, 1980, 22(3): 727–738.
[152] de Souza Martins SC, Agbulut O, Diguet N, et al. Dynamic expression of synemin isoforms in mouse embryonic stem cells and neural derivatives[J]. *BMC Cell Biol*, 2011, 12: 51.
[153] Russell MA. Synemin redefined: multiple binding partners results in multifunctionality[J]. *Front Cell Dev Biol*, 2020, 8: 159.
[154] Sun N, Critchley DR, Paulin D, et al. Human α-synemin interacts directly with vinculin and metavinculin[J].

*Biochem J*, 2008, 409(3): 657–667.

[155] Sun N, Critchley DR, Paulin D, et al. Identification of a repeated domain within mammalian α-synemin that interacts directly with talin[J]. *Exp Cell Res*, 2008, 314(8): 1839–1849.

[156] Mizuno Y, Thompson TG, Guyon JR, et al. Desmuslin, an intermediate filament protein that interacts with α-dystrobrevin and desmin[J]. *Proc Natl Acad Sci U S A*, 2001, 98(11): 6156–6161.

[157] Bhosle RC, Michele DE, Campbell KP, et al. Interactions of intermediate filament protein synemin with dystrophin and utrophin[J]. *Biochem Biophys Res Commun*, 2006, 346(3): 768–777.

[158] Maggi L, Mavroidis M, Psarras S, et al. Skeletal and cardiac muscle disorders caused by mutations in genes encoding intermediate filament proteins[J]. *Int J Mol Sci*, 2021, 22(8): 4256.

[159] Gerace L, Blobel G. The nuclear envelope lamina is reversibly depolymerized during mitosis[J]. *Cell*, 1980, 19(1): 277–287.

[160] Gerace L, Blum A, Blobel G. Immunocytochemical localization of the major polypeptides of the nuclear pore complex-lamina fraction. Interphase and mitotic distribution[J]. *J Cell Biol*, 1978, 79(2 Pt 1): 546–566.

[161] Turgay Y, Eibauer M, Goldman AE, et al. The molecular architecture of lamins in somatic cells[J]. *Nature*, 2017, 543(7644): 261–264.

[162] Tenga R, Medalia O. Structure and unique mechanical aspects of nuclear lamin filaments[J]. *Curr Opin Struct Biol*, 2020, 64: 152–159.

[163] Odell J, Lammerding J. Lamins as structural nuclear elements through evolution[J]. *Curr Opin Cell Biol*, 2023, 85: 102267.

[164] Taniura H, Glass C, Gerace L. A chromatin binding site in the tail domain of nuclear lamins that interacts with core histones[J]. *J Cell Biol*, 1995, 131(1): 33–44.

[165] Ahn J, Lee J, Jeong S, et al. Beta-strand-mediated dimeric formation of the Ig-like domains of human lamin A/C and B1[J]. *Biochem Biophys Res Commun*, 2021, 550: 191–196.

[166] Machowska M, Piekarowicz K, Rzepecki R. Regulation of lamin properties and functions: does phosphorylation do it all?[J]. *Open Biol*, 2015, 5(11): 150094.

[167] Jeong S, Ahn J, Jo I, et al. Cyclin-dependent kinase 1 depolymerizes nuclear lamin filaments by disrupting the head-to-tail interaction of the lamin central rod domain[J]. *J Biol Chem*, 2022, 298(9): 102256.

[168] Lammerding J, Schulze PC, Takahashi T, et al. Lamin A/C deficiency causes defective nuclear mechanics and mechanotransduction[J]. *J Clin Invest*, 2004, 113(3): 370–378.

[169] Donnaloja F, Carnevali F, Jacchetti E, et al. Lamin A/C mechanotransduction in laminopathies[J]. *Cells*, 2020, 9(5): 1306.

[170] Santini GT, Shah PP, Karnay A, et al. Aberrant chromatin organization at the nexus of laminopathy disease pathways[J]. *Nucleus*, 2022, 13(1): 302–314.

[171] Evangelisti C, Rusciano I, Mongiorgi S, et al. The wide and growing range of lamin B-related diseases: from laminopathies to cancer[J]. *Cell Mol Life Sci*, 2022, 79(2): 126.

[172] Röber RA, Weber K, Osborn M. Differential timing of nuclear lamin A/C expression in the various organs of the mouse embryo and the young animal: a developmental study[J]. *Development*, 1989, 105(2): 365–378.

[173] Harborth J, Elbashir SM, Bechert K, et al. Identification of essential genes in cultured mammalian cells using small interfering RNAs[J]. *J Cell Sci*, 2001, 114(Pt 24): 4557–4565.

[174] Kim Y, Sharov AA, McDole K, et al. Mouse B-type lamins are required for proper organogenesis but not by embryonic stem cells[J]. *Science*, 2011, 334(6063): 1706–1710.

[175] Kim Y, Zheng X, Zheng Y. Proliferation and differentiation of mouse embryonic stem cells lacking all lamins[J]. *Cell Res*, 2013, 23(12): 1420–1423.

[176] Reddy S, Comai L. Lamin A, farnesylation and aging[J]. *Exp Cell Res*, 2012, 318(1): 1–7.

[177] Lattanzi G, Columbaro M, Mattioli E, et al. Pre-Lamin A processing is linked to heterochromatin organization[J]. *J Cell Biochem*, 2007, 102(5): 1149–1159.

[178] Swift J, Ivanovska IL, Buxboim A, et al. Nuclear lamin-A scales with tissue stiffness and enhances matrix-directed differentiation[J]. *Science*, 2013, 341(6149): e1240104.

[179] Wang M, Ivanovska I, Vashisth M, et al. Nuclear mechanoprotection: from tissue atlases as blueprints to distinctive regulation of nuclear lamins[J]. *APL Bioeng*, 2022, 6(2): 021504.

[180] Urciuoli E, Peruzzi B. The paradox of nuclear lamins in pathologies: apparently controversial roles explained by tissue-specific mechanobiology[J]. *Cells*, 2022, 11(14): 2194.

[181] Owens DJ, Fischer M, Jabre S, et al. Lamin mutations cause increased YAP nuclear entry in muscle stem cells[J]. *Cells*, 2020, 9(4): 816.

[182] Eriksson M, Brown WT, Gordon LB, et al. Recurrent *de novo* point mutations in lamin A cause Hutchinson-Gilford progeria syndrome[J]. *Nature*, 2003, 423(6937): 293–298.

[183] De Sandre-Giovannoli A, Bernard R, Cau P, et al. Lamin a truncation in Hutchinson-Gilford progeria[J]. *Science*, 2003, 300(5628): 2055.

[184] Cisneros B, García-Aguirre I, De Ita M, et al. Hutchinson-Gilford progeria syndrome: cellular mechanisms and therapeutic perspectives[J]. *Arch Med Res*, 2023, 54(5): 102837.

[185] Primmer SR, Liao C, Kummert OMP, et al. Lamin A to Z in normal aging[J]. *Aging (Albany NY)*, 2022, 14(20): 8150–8166.

[186] Ahn J, Lee J, Jeong S, et al. Structural basis for the interaction between unfarnesylated progerin and the Ig-like domain of lamin A/C in premature aging disorders[J]. *Biochem Biophys Res Commun*, 2022, 637: 210–217.

[187] Dubik N, Mai S. Lamin A/C: function in normal and tumor cells[J]. *Cancers (Basel)*, 2020, 12(12): 3688.
[188] Guelen L, Pagie L, Brasset E, et al. Domain organization of human chromosomes revealed by mapping of nuclear lamina interactions[J]. *Nature*, 2008, 453(7197): 948–951.
[189] Maske CP, Hollinshead MS, Higbee NC, et al. A carboxyl-terminal interaction of lamin B1 is dependent on the CAAX endoprotease Rce1 and carboxymethylation[J]. *J Cell Biol*, 2003, 162(7): 1223–1232.
[190] Malhas A, Lee CF, Sanders R, et al. Defects in lamin B1 expression or processing affect interphase chromosome position and gene expression[J]. *J Cell Biol*, 2007, 176(5): 593–603.
[191] Reilly A, Philip Creamer J, Stewart S, et al. Lamin B1 deletion in myeloid neoplasms causes nuclear anomaly and altered hematopoietic stem cell function[J]. *Cell Stem Cell*, 2022, 29(4): 577–592.e8.
[192] Matias I, Diniz LP, Damico IV, et al. Loss of lamin-B1 and defective nuclear morphology are hallmarks of astrocyte senescence *in vitro* and in the aging human hippocampus[J]. *Aging Cell*, 2022, 21(1): e13521.
[193] Kim Y. The impact of altered lamin B1 levels on nuclear lamina structure and function in aging and human diseases[J]. *Curr Opin Cell Biol*, 2023, 85: 102257.
[194] Goulielmos G, Gounari F, Remington S, et al. Filensin and phakinin form a novel type of beaded intermediate filaments and coassemble de novo in cultured cells[J]. *J Cell Biol*, 1996, 132(4): 643–655.
[195] Chaves JM, Gupta R, Srivastava K, et al. Human alpha A-crystallin missing N-terminal domain poorly complexes with filensin and phakinin[J]. *Biochem Biophys Res Commun*, 2017, 494(1-2): 402–408.
[196] Oka M, Kudo H, Sugama N, et al. The function of filensin and phakinin in lens transparency[J]. *Mol Vis*, 2008, 14: 815–822.
[197] Liu Q, Wang K, Zhu S. A novel p. G112E mutation in BFSP2 associated with autosomal dominant pulverulent cataract with sutural opacities[J]. *Curr Eye Res*, 2014, 39(10): 1013–1019.
[198] Merdes A, Brunkener M, Horstmann H, et al. Filensin: a new vimentin-binding, polymerization-competent, and membrane-associated protein of the lens fiber cell[J]. *J Cell Biol*, 1991, 115(2): 397–410.
[199] Tashiro M, Nakamura A, Kuratani Y, et al. Effects of truncations in the N- and C-terminal domains of filensin on filament formation with phakinin in cell-free conditions and cultured cells[J]. *FEBS Open Bio*, 2023, 13(11): 1990–2004.
[200] Tapodi A, Clemens DM, Uwineza A, et al. BFSP1 C-terminal domains released by post-translational processing events can alter significantly the calcium regulation of AQP0 water permeability[J]. *Exp Eye Res*, 2019, 185: 107585.
[201] Merdes A, Gounari F, Georgatos SD. The 47-kD lens-specific protein phakinin is a tailless intermediate filament protein and an assembly partner of filensin[J]. *J Cell Biol*, 1993, 123(6 Pt 1): 1507–1516.
[202] Alizadeh A, Clark JI, Seeberger T, et al. Targeted genomic deletion of the lens-specific intermediate filament protein CP49[J]. *Invest Ophthalmol Vis Sci*, 2002, 43(12): 3722–3727.
[203] Wiche G. Plectin in health and disease[J]. *Cells*, 2022, 11(9): 1412.
[204] Gao K, Gao Z, Xia M, et al. Role of plectin and its interacting molecules in cancer[J]. *Med Oncol*, 2023, 40(10): 280.
[205] Ortega E, Manso JA, Buey RM, et al. The structure of the plakin domain of plectin reveals an extended rod-like shape[J]. *J Biol Chem*, 2016, 291(36): 18643–18662.
[206] Wiche G, Winter L. Plectin isoforms as organizers of intermediate filament cytoarchitecture[J]. *Bioarchitecture*, 2011, 1(1): 14–20.
[207] Kiritsi D, Tsakiris L, Schauer F. Plectin in skin fragility disorders[J]. *Cells*, 2021, 10(10): 2738.
[208] Kostan J, Gregor M, Walko G, et al. Plectin isoform-dependent regulation of keratin-integrin α6β4 anchorage *via* $Ca^{2+}$/calmodulin[J]. *J Biol Chem*, 2009, 284(27): 18525–18536.
[209] Wiche G. Plectin-mediated intermediate filament functions: why isoforms matter[J]. *Cells*, 2021, 10(8): 2154.
[210] Andrä K, Kornacker I, Jörgl A, et al. Plectin-isoform-specific rescue of hemidesmosomal defects in plectin (-/-) keratinocytes[J]. *J Invest Dermatol*, 2003, 120(2): 189–197.
[211] Foisner R, Leichtfried FE, Herrmann H, et al. Cytoskeleton-associated plectin: *in situ* localization, *in vitro* reconstitution, and binding to immobilized intermediate filament proteins[J]. *J Cell Biol*, 1988, 106(3): 723–733.
[212] Walko G, Castañón MJ, Wiche G. Molecular architecture and function of the hemidesmosome[J]. *Cell Tissue Res*, 2015, 360(3): 529–544.
[213] Son S, Baek A, Lee JH, et al. Autophagosome-lysosome fusion is facilitated by plectin-stabilized actin and keratin 8 during macroautophagic process[J]. *Cell Mol Life Sci*, 2022, 79(2): 95.
[214] Andrä K, Nikolic B, Stöcher M, et al. Not just scaffolding: plectin regulates actin dynamics in cultured cells[J]. *Genes Dev*, 1998, 12(21): 3442–3451.
[215] Abrahamsberg C, Fuchs P, Osmanagic-Myers S, et al. Targeted ablation of plectin isoform 1 uncovers role of cytolinker proteins in leukocyte recruitment[J]. *Proc Natl Acad Sci U S A*, 2005, 102(51): 18449–18454.
[216] Kowalczyk AP, Green KJ. Structure, function, and regulation of desmosomes[J]. *Prog Mol Biol Transl Sci*, 2013, 116: 95–118.
[217] Moazzen H, Bolaji MD, Leube RE. Desmosomes in cell fate determination: from cardiogenesis to cardiomyopathy[J]. *Cells*, 2023, 12(17): 2122.
[218] Wilson KL, Foisner R. Lamin-binding proteins[J]. *Cold Spring Harb Perspect Biol*, 2010, 2(4): a000554.
[219] de Leeuw R, Gruenbaum Y, Medalia O. Nuclear lamins: thin filaments with major functions[J]. *Trends Cell Biol*,

2018, 28(1): 34–45.
[220] Lityagina O, Dobreva G. The LINC between mechanical forces and chromatin[J]. *Front Physiol*, 2021, 12: 710809.
[221] Meqbel BRM, Gomes M, Omer A, et al. LINCing senescence and nuclear envelope changes[J]. *Cells*, 2022, 11(11): 1787.
[222] Belaadi N, Guilluy C. Life outside the LINC complex - do SUN proteins have LINC-independent functions?[J]. *Bioessays*, 2024, 46(8): 2400034.
[223] Marcelot A, Worman HJ, Zinn-Justin S. Protein structural and mechanistic basis of progeroid laminopathies[J]. *FEBS J*, 2021, 288(9): 2757–2772.
[224] Wilhelmsen K, Litjens SHM, Kuikman I, et al. Nesprin-3, a novel outer nuclear membrane protein, associates with the cytoskeletal linker protein plectin[J]. *J Cell Biol*, 2005, 171(5): 799–810.
[225] Roux KJ, Crisp ML, Liu Q, et al. Nesprin 4 is an outer nuclear membrane protein that can induce kinesin-mediated cell polarization[J]. *Proc Natl Acad Sci U S A*, 2009, 106(7): 2194–2199.
[226] Morimoto A, Shibuya H, Zhu X, et al. A conserved KASH domain protein associates with telomeres, SUN1, and dynactin during mammalian meiosis[J]. *J Cell Biol*, 2012, 198(2): 165–172.
[227] Horn HF, Kim DI, Wright GD, et al. A mammalian KASH domain protein coupling meiotic chromosomes to the cytoskeleton[J]. *J Cell Biol*, 2013, 202(7): 1023–1039.
[228] Garner KEL, Salter A, Lau CK, et al. The meiotic LINC complex component KASH5 is an activating adaptor for cytoplasmic dynein[J]. *J Cell Biol*, 2023, 222(5): e202204042.
[229] Okumura W, Tadahira K, Kozono T, et al. Jaw1/LRMP is associated with the maintenance of Golgi ribbon structure[J]. *J Biochem*, 2023, 173(5): 383–392.
[230] Haque F, Lloyd DJ, Smallwood DT, et al. SUN1 interacts with nuclear lamin A and cytoplasmic nesprins to provide a physical connection between the nuclear lamina and the cytoskeleton[J]. *Mol Cell Biol*, 2006, 26(10): 3738–3751.
[231] Thoma H, Grünewald L, Braune S, et al. SUN4 is a spermatid type Ⅱ inner nuclear membrane protein that forms heteromeric assemblies with SUN3 and interacts with lamin B3[J]. *J Cell Sci*, 2023, 136(7): jcs260155.
[232] Korfali N, Wilkie GS, Swanson SK, et al. The nuclear envelope proteome differs notably between tissues[J]. *Nucleus*, 2012, 3(6): 552–564.
[233] Berk JM, Wilson KL. Simple separation of functionally distinct populations of lamin-binding proteins[J]. *Methods Enzymol*, 2016, 569: 101–114.
[234] Simon DN, Wilson KL. Partners and post-translational modifications of nuclear lamins[J]. *Chromosoma*, 2013, 122(1-2): 13–31.
[235] Herrada I, Bourgeois B, Samson C, et al. Purification and structural analysis of LEM-domain proteins[J]. *Methods Enzymol*, 2016, 569: 43–61.
[236] Barton LJ, Soshnev AA, Geyer PK. Networking in the nucleus: a spotlight on LEM-domain proteins[J]. *Curr Opin Cell Biol*, 2015, 34: 1–8.
[237] Martins S, Eikvar S, Furukawa K, et al. HA95 and LAP2β mediate a novel chromatin-nuclear envelope interaction implicated in initiation of DNA replication[J]. *J Cell Biol*, 2003, 160(2): 177–188.
[238] Cai M, Huang Y, Ghirlando R, et al. Solution structure of the constant region of nuclear envelope protein LAP2 reveals two LEM-domain structures: one binds BAF and the other binds DNA[J]. *EMBO J*, 2001, 20(16): 4399–4407.
[239] Caputo S, Couprie J, Duband-Goulet I, et al. The carboxyl-terminal nucleoplasmic region of MAN1 exhibits a DNA binding winged helix domain[J]. *J Biol Chem*, 2006, 281(26): 18208–18215.
[240] Balaji AK, Saha S, Deshpande S, et al. Nuclear envelope, chromatin organizers, histones, and DNA: the many achilles heels exploited across cancers[J]. *Front Cell Dev Biol*, 2022, 10: 1068347.
[241] Manda NK, Golla U, Sesham K, et al. Tuning between nuclear organization and functionality in health and disease[J]. *Cells*, 2023, 12(5): 706.
[242] Gong H, Zhou H, McKenzie GW, et al. An updated nomenclature for keratin-associated proteins (KAPs)[J]. *Int J Biol Sci*, 2012, 8(2): 258–264.
[243] Litman T, Stein WD. Ancient lineages of the keratin-associated protein (KRTAP) genes and their co-option in the evolution of the hair follicle[J]. *BMC Ecol Evol*, 2023, 23(1): 7.
[244] Matsunaga R, Abe R, Ishii D, et al. Bidirectional binding property of high glycine-tyrosine keratin-associated protein contributes to the mechanical strength and shape of hair[J]. *J Struct Biol*, 2013, 183(3): 484–494.
[245] Takahashi K, Podyma-Inoue KA, Saito M, et al. TGF-β generates a population of cancer cells residing in G1 phase with high motility and metastatic potential *via* KRTAP2-3[J]. *Cell Rep*, 2022, 40(13): 111411.
[246] Kim J, Jang J, Yang C, et al. Vimentin filament controls integrin α5β1-mediated cell adhesion by binding to integrin through its Ser38 residue[J]. *FEBS Lett*, 2016, 590(20): 3517–3525.
[247] Tong X, Coulombe PA. Keratin 17 modulates hair follicle cycling in a TN*F*α-dependent fashion[J]. *Genes Dev*, 2006, 20(10): 1353–1364.
[248] Rotty JD, Coulombe PA. A wound-induced keratin inhibits Src activity during keratinocyte migration and tissue repair[J]. *J Cell Biol*, 2012, 197(3): 381–389.
[249] Peng N, Zhang Y, Zhang X, et al. NAP1L1 is a novel microtubule-associated protein[J]. *Cytoskeleton (Hoboken)*, 2023, 80(9-10): 382–392.
